# The type of environment has a greater impact on the larval microbiota of *Anopheles arabiensis* than on the microbiota of their breeding water

**DOI:** 10.1093/femsec/fiae161

**Published:** 2024-12-18

**Authors:** Lorenzo Assentato, Louise K J Nilsson, Carl Brunius, Vilhelm Feltelius, Rasmus Elleby, Richard J Hopkins, Olle Terenius

**Affiliations:** Department of Cell and Molecular Biology, Microbiology and Immunology, Uppsala University, Box 596, SE-751 24 Uppsala, Sweden; Department of Cell and Molecular Biology, Microbiology and Immunology, Uppsala University, Box 596, SE-751 24 Uppsala, Sweden; Department of Ecology, Swedish University of Agricultural Sciences (SLU), Box 7044, SE-750 07 Uppsala, Sweden; Department of Life Sciences, Food and Nutrition Science, Chalmers University of Technology, SE-412 96 Göteborg, Sweden; VA-guiden Sverige AB, Östra Ågatan 53, 4 tr, SE-753 22 Uppsala, Sweden; VA-guiden Sverige AB, Östra Ågatan 53, 4 tr, SE-753 22 Uppsala, Sweden; Natural Resources Institute, University of Greenwich, Central Avenue, Chatham Maritime, Kent ME4 4 TB, United Kingdom; Department of Cell and Molecular Biology, Microbiology and Immunology, Uppsala University, Box 596, SE-751 24 Uppsala, Sweden

**Keywords:** SML, microbiota, *Anopheles*, breeding site, random forest, ASV

## Abstract

Mosquito larvae of the genus *Anopheles* develop entirely in water, frequently visiting the surface for air. The aquatic environment plays a key role in shaping their microbiota, but the connection between environmental characteristics of breeding sites and larval microbiota remains underexplored. This study focuses on *Anopheles arabiensis*, which inhabits the surface microlayer (SML) of breeding sites, a zone with high particle density. We hypothesized that the SML could allow us to capture the diversity of the surrounding environment, and in turn its influence on the larval microbial communities. To test this, we collected *A. arabiensis* larvae and SML samples from various breeding sites categorized by environmental features. Our results confirm that breeding site characteristics are significant drivers of the bacterial species present in mosquito larvae. Additionally, we found that the larval micro-environment selectively shapes its microbiota, highlighting a dynamic interplay between environmental and internal factors. Interestingly, specific bacterial families were associated with the presence or absence of larvae in breeding sites, suggesting potential ecological roles. These findings expand our understanding of vector-mosquito microbiota, emphasizing the importance of breeding site features in shaping larval microbial communities and providing a foundation for future research on mosquito ecology and control strategies.

## Teaser

Environmental characteristics of the breeding site are an important factor in shaping the microbiota of mosquitoes of the genus *Anopheles*.

## Introduction

Insects are exposed to an environment full of microbes, which structure symbiotic relationships with them, constituting their microbiota. Most of these bacterial species get incorporated because of progressive inputs during the life cycle of an individual, and host–microbiota interactions can be used to gain information on the insect host and its life history. This is especially true for mosquitoes, for which the microbiota conveys information because of the strong relationship between the individual mosquito and location of collection, because the larvae and pupae are confined to a single aquatic environment. For instance, in Buck et al. (2016) villages are shown to play a significant role in determining differences in microbiota composition. Similarly, a more recent paper from Schrieke et al. (2022) concludes that geography is a primary factor in influencing mosquito bacterial communities. Other works (e.g. Akorli et al. 2016) showed how seasonality also can correlate with microbial composition, together with location. Moreover, Gimonneau et al. (2014) proved that inter-location variability exists in individual larvae and carries over to the adult stage, and Girard et al. (2021) gives a good overview of microorganisms involved in mosquito attractiveness/repellence to breeding site selection, which in turn contributes to habitat selection and to further diversify microbial communities.

In addition, other mechanisms influence the composition of mosquito microbiota. Trans-stadial transfer, for example, in which microbial taxa carry over between different developmental stages in the mosquito life cycle (Lindh et al. 2008). During oviposition, behaviors like egg-smearing or just contact with the water determine a reduction of the complexity of water microbiota, and the increase of species beneficial for mosquito survival (Mosquera et al. 2023). All the listed mechanisms could contribute over successive generations of mosquitoes to further select and establish a microbial community strongly correlated to a specific mosquito genus. This is in accordance with niche construction theory, which describes environmental modification by the inhabiting organisms and their accumulation over time as an evolutionary process in its own right (Odling-Smee et al. 2013).

Mosquito larvae are filter feeders, developing in close contact with the water microbiota, which becomes both a source for nutrients and a factor shaping their symbiotic interactions. In particular, mosquitoes of the genus *Anopheles* exhibit a filter-feeding behavior in which they float on top of the water column, on the water–air interface, called the surface microlayer (SML) (Maki and Hermansson 1994). This very thin area has a gel-like consistency determined by the high abundance of dissolved particles, compared to underlying water, and the higher abundance of living bacteria involved in the production of biofilm (Wurl and Holmes 2008). The physical property of the SML determines a higher interactivity of this layer with the surrounding environment, collecting debris and organic matter that in turn are fed on by *Anopheles* larvae (Wotton et al. 1997).

Breeding sites of different types can be associated with quite different vegetation coverage, as well as different plant and animal species present in the location and other physico-chemical parameters of the water influencing mosquito detectability and location productivity (Mala and Irungu 2011, Low et al. 2016, Ouédraogo et al. 2022). Thus, we hypothesize that the SML layer in water locations from different sites might also vary significantly, influencing in turn also the mosquito microbiota composition.

This hypothesis was tested on a collection of *Anopheles arabiensis* larvae from Ethiopia. First, we focus on the nature of the breeding sites and its association with the microbiota, analyzing in detail microbial communities both in water and larval samples from different breeding site types. Then, we compared the overlapping portion of the microbial communities of water and larvae across different types of breeding sites to assess the relative abundance of important bacterial species. Finally, since mosquito presence was not uniform in the different sampling locations, we follow this up with an analysis of the correlation between water physico-chemical parameters and mosquito microbiota, to determine associations between water quality and important bacterial families.

With these aims, we shed light on an aspect of mosquito microbiota that has not yet been explored extensively, namely the relation between types of breeding sites (i.e. river fringes, marshes), especially in the case of natural sites, and the microbiota composition.

## Materials and methods

### Study area

The study area comprises 26 sampling sites of both natural and artificial origin, in the Gamo Gofa Zone, Rift Valley, in the Arba Minch Zuria and Mirab Abaya Woredas, Ethiopia (Supplementary Fig. 1E). The sites are located in rural areas at 1200 m above sea level, where transmission of malaria is seasonal or all-year round. Sampling was conducted in 2014 during a 1-month period, 6th of February to 6th of March (Supplementary Table 1). The sites represent different types of breeding sites, classified through the observation of common environmental characteristics (Supplementary Fig. 1A–D).

### Collection of surface-micro layer and larval samples

Surface microlayer water was collected using a thin stainless steel wire mesh measuring 400 cm^2^, with a mesh size of 1.25 and 0.36 mm wire diameter (Briones et al. 2008). The wire mesh was first sprayed with 95% ethanol and flamed before use, to sterilize it. Subsequently, the mesh was placed gently on top of the water surface and lifted while kept in a horizontal position to collect through surface tension a thin film of surface water. The collection was repeated twice, in different areas in the water body if size allowed. Each dip collected 20–22 ml of water that was then pooled in a 50 ml sterile-capped container. Samples were filtered with Whatman Grade 1 Qualitative Filter Paper (GE Health-care, Uppsala, Sweden) to remove particles from the water (11 µm particle retention), samples were sent to Uppsala, Sweden, for further handling.

Out of 26 sites, 16 were positive for larval presence (Supplementary Table 1). Mosquito larvae were collected using a 293 ml dipper. Five dips or more were made at each site at various locations within the site. The collected larvae were identified as either *Anopheles* or non-*Anopheles* and as either early (first or second) or late (third or fourth) instar in the dipper. *Anopheles* larvae were stored in Eppendorf tubes with 78% ethanol. The larvae were transported to Uppsala, Sweden and in the lab identified as *A. arabiensis* using morphological identification and Polymerase Chain Reaction (PCR) according to Scott et al. (1993).

### Recording of physico-chemical parameters

Water physico-chemical parameters were measured at each site included in the study, with a mix of *in situ* or in laboratory measurements. Each measurement was repeated three times, and the averaged result was considered for each site.

Measurements for temperature (°C ± 0.01), pH (±0.01), electrical conductivity (EC) (mS/cm ± 2% full scale), and total dissolved solids (TDSs) (ppt ± 2% full scale) were conducted *in situ* with a portable meter HI-991301 from HANNA instruments. Following the manufacturer's instructions, measurements were taken at three different spots in the collection site.

Measurements for dissolved oxygen (ppm 20°C ± 1.5%) were also conducted *in situ*, using a portable meter HI-9146 from HANNA instruments. In this case, the measurement value has been registered while holding the meter submerged in water and spinning it in a small circle, to ensure that the oxygen-depleted membrane surface would be replenished.

Measurements of turbidity (FTU ± 0.5) and phosphates low range (PO_4_^3−^ mg/l ± 0.4) were conducted in a lab. Turbidity was measured with a HI-93703 meter from HANNA instruments. Only one sample from each site was taken, before conducting any dipping or water analysis so that the samples were undisturbed. The measurement was conducted according to the manufacturer's instructions. Phosphate concentration was measured with a photometer HI-83200 from HANNA instruments, with a wavelength of 610 nm, using the same water samples used for turbidity analysis. In case the turbidity was high, active carbon was added to the sample prior to filtering.

### DNA extraction and PCR amplification

DNA extraction was performed on individual late instar larvae using QIAamp DNA mini kit (Qiagen) according to the manufacturer's protocol. Extracted DNA was diluted in 75 µl nuclease-free water and concentrations were measured using a NanoDrop ND-1000 (Saveen & Werner). For the SML samples, two replicates were used for each site. Two sites identified as “O” and “R” did not yield enough DNA, bringing the total to 48 SML samples. For the larval samples, 9 sites out of 16 were selected. The selection was based on the following criteria: Locations where *Anopheles* larvae were found; locations where at least six larval samples could be collected. Lastly, we made sure to select, when possible, at least two locations per type of breeding site, if those met the previous two conditions. These 9 sites accounted for a total of 54 samples. PCR amplification of the extracted DNA was performed using a two-step method as previously described (Buck et al. 2016) to produce barcoded bacterial 16S rRNA gene amplicons for sequencing. Along with the samples in this study, a blank sample was included to act as a negative control, and it followed through all the steps of the processing.

All PCRs were performed using Illustra PuReTaq Ready-To-Go PCR Beads (GE Healthcare). The first step PCR used 0.4–0.8 µM per reaction of the primers 341F (5′-CCTACGGGNGGCWGCAG-3′) and 805R (5′-GACTACHVGGGTATCTAATCC-3′) that target the V3–V4 region of the bacterial 16S rRNA gene. Per PCR reaction, 50–70 ng DNA was used as templates. The PCR program had an initial denaturation step at 95°C for 5 min, 30 cycles of [95°C for 40 s, 53°C for 40 s, and 72°C for 60 s], and a final elongation step at 72°C for 7 min. The PCR products of the first step PCR were analyzed by microchip electrophoresis by MCE-202 MultiNA (Shimadzu) and diluted in nuclease-free water to a concentration of 0.1–1 ng/µl. In the second step PCR, 1 µl of the diluted PCR product was used as a template. The second step PCR was performed with 50 different barcoding primer pairs (0.8 µM per primer per reaction) to be able to pool and sequence 50 samples per library (Sinclair et al. 2015). The second step PCR program was the same as the first but using only 10 cycles. The PCR products of the second step PCR were also analyzed by microchip electrophoresis by MCE-202 MultiNA (Shimadzu). The products from the second step PCR were pooled together, 50 differently barcoded samples per pool and 60 ng per sample. Each pool was purified using Illustra GFX PCR DNA and Gel Band Purification Kit (GE Healthcare) and eluted in 50 µl nuclease-free water.

### Library preparation and MiSeq sequencing

Sequencing libraries and MiSeq sequencing were prepared and performed by the SNP&SEQ Technology Platform in Uppsala, Sweden (www.sequencing.se). Libraries were prepared from ∼10 ng of DNA using the ThruPLEX-FD Prep Kit (R40048-08, Rubicon Genomics) according to the manufacturer's instructions. Briefly, the DNA fragments were end-repaired followed by ligation of stem-loop adaptors. The 3′ ends of the genomic DNA were then extended and amplified to include indexes, followed by additional library amplification for 8 cycles of PCR. The libraries were then purified using AMPure XP beads. The quality of the library was evaluated using the 2200 TapeStation system (Agilent Technologies) and the D1000 Analysis ScreenTape assay. The adapter-ligated fragments were quantified by qPCR using the library quantification kit for Illumina (KAPA Biosystems) on a StepOnePlus instrument (Applied Biosystems/Life technologies) prior to cluster generation and sequencing. Sequencing was performed by paired-end sequencing with 300 bp read length and the “v3” chemistry using the MiSeq system (Illumina) according to the manufacturer's protocols.

### Raw sequence data processing

Data was archived for a period of around 8 years waiting for further bioinformatic processing.

Samples were demultiplexed according to the barcode pairs using Cutadapt (v. 4.1), searching for barcode pairs (–pair-adapters) in both forward and reverse orientation, allowing for 0 errors since the barcodes used for multiplexing are relatively short (seven nucleotides). Furthermore, still using Cutadapt, primers were removed from individual samples according to the primer pair used during the amplification step (341F-805R), allowing for the use of IUPAC wildcards, with a default error threshold of 10% of the total primer length (respectively, 1 error for the forward primer that is 17 nucleotides long, 2 errors for the reverse, that is 21 nucleotides), and searching for both forward and reverse orientation. A further filter on length was implemented, where the sequences allowed to pass the primer removal step needed to be between 200 and 300 nucleotides long, otherwise the whole pair was discarded, this is to limit the effect of very short reads and non-specific amplification on the downstream processing and merging of the sequences. The corresponding files (cleaned from barcodes and primers) are available on the European Nucleotide Archive (ENA) under the accession number: PRJEB76915.

After removal of barcodes and primers, an initial quality control was performed using FastQC (v. 0.11.9), whose results have been summarized with MultiQC (v. 1.13). After this initial quality control, the samples were processed as with the use of Trim Galore (v. 0.6.7) using the default option, and that operated a variable threshold trimming, removing low quality bases from reads, and additionally searched for sequences corresponding to standard illumina adapters, which were eventually removed. This initial processing of raw sequencing data yielded a total amount of 1 076 266 reads divided among 102 samples.

### ASVs inference and filtering

After trimming, samples were processed using the DADA2 package (Callahan et al. 2016) (v. 1.26.0) in R (v. 4.2.0), which filters reads using standard parameters. However, since the reads had been trimmed according to quality already, no further truncation was made. An error model of the run was created, and amplicon sequence variants (ASVs) were inferred with a pooling algorithm, to ease the resolution of rare variants by sharing information across samples (Callahan et al. 2016).

Reads were merged, and the resulting ASVs were filtered by length, in the range 410–470 nt, to avoid the retention of shorter ASVs that might have resulted from non-specific priming, and lastly chimeric sequences were removed. A summary table of the read processing can be found in the supplementary material (Supplementary Table 2), after processing 443 863 reads were kept, divided among 102 samples.

Following processing through DADA2, taxonomic assignment was performed through the use of the DECIPHER package (v. 2.26.0) with IDTAXA (Murali et al. 2018), on both strands, with a confidence threshold of 60%, sequences were identified through the use of the modified SILVA SSU (release 138) (Quast et al. 2013) training set provided by DECIPHER. For a detailed description of how the training set is constructed, see http://www2.decipher.codes/ClassifyOrganismsFAQ.html.

In short, a total of 11 different phyla, 17 classes, 43 orders, 65 families, and 117 genera of bacteria were identified. At the respective level, the percentages of unidentified taxa were: phylum 4.87%, class 5.13%, order 7.57%, family 12.27%, genus 42.4%, for details c.f. Supplementary Table 3.

After obtaining taxonomical classification, a count table and a taxonomy table were generated, and imported into the Phyloseq package (McMurdie and Holmes 2013) (v. 1.42.0) for filtering eventually unclassified sequences at the phylum level, as well as eventual chloroplast and mitochondrial contaminants. A further filtering step based on prevalence was implemented, with the aim of improving the downstream analysis by removing rare taxa that might be a result of contamination (Cao et al. 2021); taxa were removed if they were present in less than 3% of the samples.

Moreover, in the negative control sample some ASVs were identified, and using the “Decontam” package we labeled some of them as “possible” contaminants. We opted to not remove those ASVs, since the reliability of a single negative control is not high, however we present a list of them in supplementary material (supplementary_data-list_1.docx).

### Sample coverage assessment and normalization of count data

The samples were processed following the indications for working with compositional data as described by Aitchison (CODA) (Gloor et al. 2017), and rarefaction was not performed to not waste useful data, due to the low sequencing depth (McMurdie and Holmes 2014).

An overview of how the samples are distributed across different types of breeding sites can be found in Supplementary Fig. 2. Sample coverage was assessed using the iNEXT package (Hsieh et al. 2016) (v. 3.0.0).

The coverage approach was chosen as a measure of completeness of a sample, to ensure accurate description of our microbial communities that would not be based on the observation of the tangents of the sample curves in rarefaction plots alone (Chiu 2023).

The results (Supplementary Table 4) highlight the effect of low number of sequences on coverage. The way coverage is estimated has been implemented in a work by Chao and Jost as a perfectioned version of the Turing's estimator with a smaller mean squared error (Chao and Jost 2012). It is an estimator of how complete a sample is; for instance, a sample at 98% coverage is likely missing 2% of the species from that community.

The coverage was then calculated separately for each single set of data: larval and SML samples, since they are expected to contain different species, not to be overlapping. Afterward, a threshold at which to exclude low-completeness samples was chosen.

For the water samples, a threshold was set at 98% coverage (which corresponds to a depth of 1453 reads), and all the samples with a lower coverage than that were excluded from the analysis. The threshold was set to this value to not cause the removal of too many samples from the dataset.

Seven samples were removed: A1, C2, E1, F1, W2, Y2, T2. Furthermore, Y1 was also removed because site Y was the only representative of “natural pond”, to not retain only a single sample from this breeding site type, the total number of SML samples kept was 40.

Concerning the larval samples, we would have opted to maintain the same threshold. However, to retain enough statistical power we aimed to keep six larval samples per location. A threshold of 98% coverage would have removed a portion of the samples from some locations, rendering the dataset unbalanced and possibly invalidating our statistical analysis. For that reason, we opted for a threshold of 95% coverage (corresponding for this dataset to 623 reads), the highest it could be without causing the removal of samples. For that reason, it is necessary to acknowledge that the SML dataset might be approximately three percentage points more “well described” when compared to the larval one.

To further assess the possibility of not explaining enough of the microbial complexity of the samples, rarefaction/extrapolation curves have been produced with the use of iNEXT (Supplementary Fig. 3). The curves have been computed not only considering the number of observed species, but also for Shannon index, a frequently used metric of alpha diversity, to determine if the chosen thresholds would hinder future comparisons between samples. However, that is not the case, with most of the samples reaching a plateau quite early, especially in the case of Shannon diversity.

Both the groups of samples were normalized to reduce the effect of varying sample depth, using “centred log ratio” (CLR) (pseudocount = 1), and “robust CLR” (RCLR) normalization as implemented in the “decostand” function of the Vegan package. The reason to adopt two different normalization techniques is that we find the second one to be a more useful implementation when in use with heatmaps, making the interpretation of plots much clearer, while we found the CLR to be less sensitive to “double zero” problem and robust to use with ordinations.

### Statistical analyses in R

Post-normalization, principal component analysis (PCA) was used to assess the differences in beta diversity between the samples for breeding site type or presence of larvae in a location. The PCA was performed on clr-normalized data and the distance measure is known as “aitchison” distance, both the calculations and the plots have been performed with the use of the factoextra package (Extract and Visualize the Results of Multivariate Data Analyses) (v. 1.0.7).

Alpha diversity has instead been calculated for two different indexes, Shannon and Simpson, with the use of the function “diversity” from the Vegan package; subsequently, the box_plots were drawn with the use of ggplot2 (v. 3.4.3).

Canonical correspondence analysis was performed with the “cca” function from the Vegan package, on absolute counts, as the analysis is invariant to rescaling. The cross-correlation matrix for the choice of which variables to include in the canonical correspondence analysis (CCA) was calculated separately with the use of CCA package, function “matcor” (González et al. 2008) (v. 1.2.2).

Indicator species analysis was performed to find species that were highly diagnostic for presence of mosquitoes, using the function “multipatt” from the package indicspecies (Cáceres and Legendre 2009) (v. 1.7.14), only associations to singleton of groups were considered, the selected ASVs underwent 999 permutations and results were considered significant if below a *P*-value of .05.

Multivariate modelling was performed to predict the breeding site or other labels from the ASV profile and explore the features driving this association. Using the MUVR package (Shi et al. 2019) (v. 0.0.975), employing the random forest (RF) algorithm within a repeated double cross-validation (rdCV) scheme. The repeated training and validation of the model was performed 20 times, because from our tests an increase did not yield better classification performance on this dataset. About employed parameters, 90% of the variables were retained for iteration and the outer segments set to 6. Three different models are produced: “min”, “mid”, and “max” depending on how many variables are deemed as significant. The “min” model uses the least number of significant variables to give an accurate classification, while the “max” model uses the max number of variables it can before classification performance starts to degrade with the inclusion of more variables. Also, a permutation of the modelling effort was employed to determine its statistical significance, the whole model was built multiple times (*n* = 100) on randomly assigned variable labels to build a population of “null” models to compare results to.

To test the significance of difference between breeding sites in the beta-diversity plots, PERMANOVA was used, with the implementation present in the “adonis2” function (permutations = 999) from the Vegan package. Similarly, homogeneity of variance was tested with the “betadisper” and “permutest” functions from the Vegan package (permutations = 999) to verify the adherence to PERMANOVA assumptions. In the occasion of a significant difference of the groups dispersion, pairwise comparisons of group mean dispersions was performed measuring Tukey's honest significant differences to assess if individual groups were influencing the result. Kruskal–Wallis test of significance used with alpha diversity calculations was performed using the “kruskal.test” function from R. Significance test for physico-chemical parameters in the CCA plot was performed using the “anova” function from R.

Pairwise distances of sites were calculated according to the geographical coordinates. Coordinates were registered in degrees, minutes, and seconds format, which was converted to degrees and pairwise distances were computed using the function “distance” from the GeoPy package (v. 2.4.1) in Python (v. 3.9.16).

To obtain a more detailed classification for ASVs which were not classified by DECIPHER, nucleotide BLAST was employed, with standard parameters and using as a reference database the rRNA database “16S ribosomal RNA sequences (Bacteria and Archea)”.

Compositional profiles, and other visualizations and plots have been produced using a mix of graphical packages in R. Ggplot2 (v. 3.4.3) was used in almost every plot, ggvenn (v. 0.1.10) was used to draw venn diagrams, ggsci (v. 3.0.0) and ggpubr (v. 0.6.0) was used to create physico-chemical parameters plots and to arrange the ordination plots, ComplexHeatmap (v. 2.14.0) has been used to plot the abundance heatmaps and perform hierarchical clustering on samples or ASVs.

## Results

### Influence of type of breeding site on bacterial composition

Our first goal was assessing the possibility that the type of breeding site might influence the microbiota in SML and larval samples. This possibility was analyzed using a PCA that highlighted how closely together the samples clustered, based on the underlying microbial composition.

**Figure 1.**
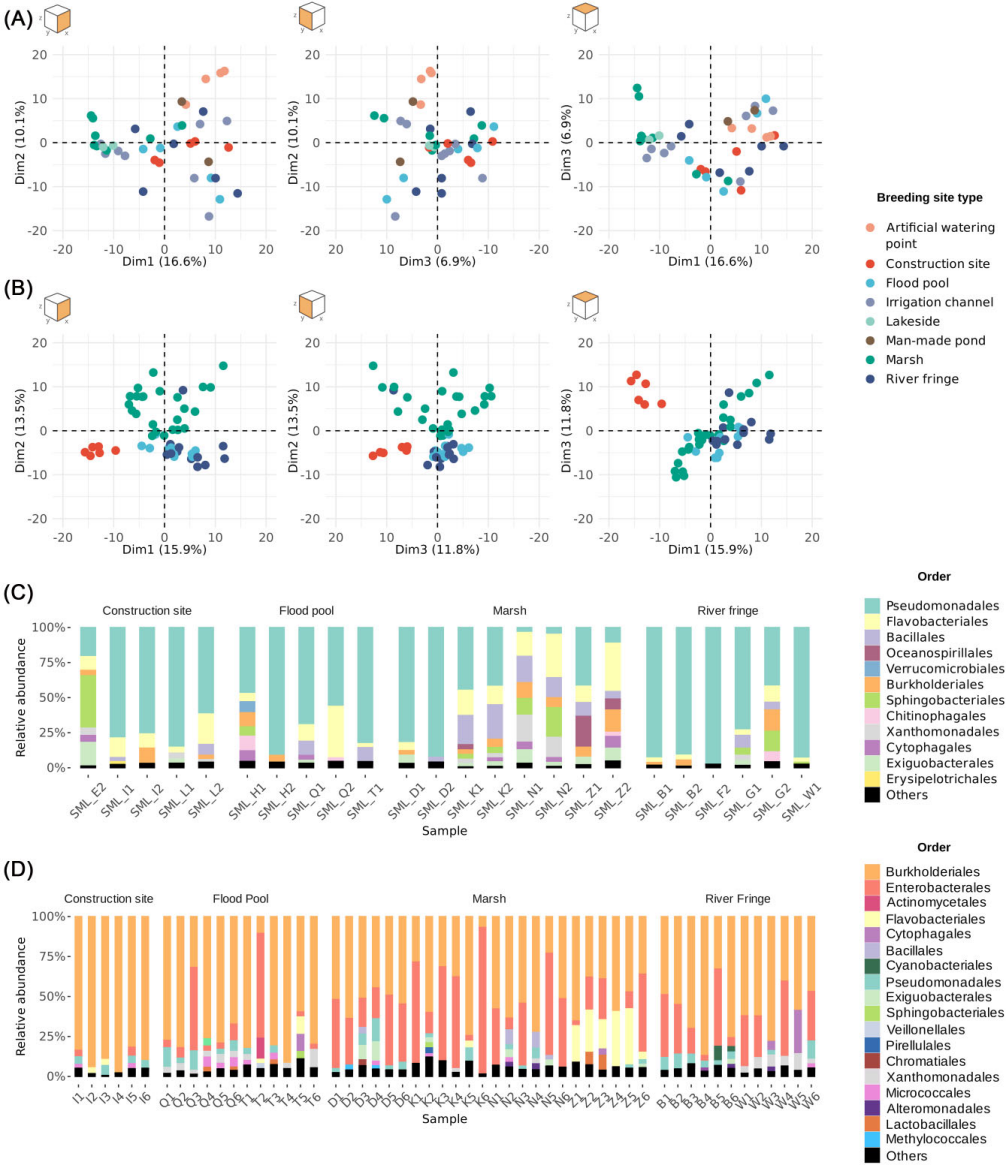
PCA on SML (A) and larval samples (B). The first three PCs have been drawn in pairs, to represent the samples in a three-dimensional space. Compositional profiles for SML (C) and larval samples (D) showing the relative abundance of different orders per sample.

The PCA plots of the water samples (*n* = 40) (Fig. 1A) shows no clear clustering behavior in the first three principal components (PCs), the total variance amount of 33.9% suggests that the dataset is characterized by a high variance that is difficult to summarize with an ordination, and that many taxa might be shared among samples. The samples frequently overlap with each other, suggesting a small influence of the type of breeding site in determining a difference in microbiota composition across different SML samples. When testing the statistical significance, however, the effect of the breeding site type on the microbial composition of the samples is significant (PERMANOVA: *R*^2^ = 0.27, *P* = .001), this suggests that the ordination might be influenced by the small sample size for each type, with an effect on the detectability of the clustering effect.

In the case of the larval samples (*n* = 56) (Fig. 1B), the PCA shows instead how samples from the same type of breeding site tend to cluster together, the total variance expressed from the first three PCs amounts to 41.2%, indicating a more complete overview and the presence of more clearly distinguished samples. Moreover, the results suggest the existence of differences in the microbial composition of “construction site” samples in particular, and a higher variability of samples belonging to the “marsh” type, probably due to the presence of cattle in some of the sites, where other marsh sites were undisturbed. Moreover, the two groups “river fringe” and “flood pool” are overlapping on multiple PCs, indicating the presence of similar microbial composition between these two groups of samples, when compared to the other locations. Again, the effect was tested with a PERMANOVA and shown to be significant (PERMANOVA: *R*^2^ = 0.27, *P* = .001). Since the PERMANOVA was significant in both instances, a test for homogeneity of variance was used to assess how reliable the result was. The homogeneity of variance shows in the case of SML data a non-significant result (*F* = 2.28, *P* = .052) indicating that the groups have a comparable mean dispersion, and thus the conclusions from the PERMANOVA test can be trusted. However, when considering larval samples, the result was significant (*F* = 21.30, *P* = .003), indicating that the groups have a significantly different dispersion and the PERMANOVA results might be biased. When comparing the pairwise mean dispersions for larval groups using Tukey's honest dispersion (Table 1), we found that the main difference was probably to be found in the different sample size between groups (construction site = 6; flood pool = 12; river fringe = 12; marsh = 24) . It is evident that in the case of larval samples the use of PERMANOVA as a statistical tool is not enough to allow us to draw conclusions.

**Table 1.** Result of pairwise Tukey's honest dispersion calculation for larval groups. The “diff” column states the difference in observed means, “lwr” and “upr” are respectively the lower and upper end point of the interval, “p-adj” gives the adjuster *P*-value for each pairing. Significance threshold is set at 0.05, significant results have been labeled with an asterisk.

Group	diff	lwr	upr	p-adj
Flood Pool-Construction Site	2.579884	−1.4493503	6.609119	0.3336456
Marsh-Construction Site	5.715523	2.0 373523	9.393694	0.0007735*
River Fringe-Construction Site	4.324851	0.2 956169	8.354086	0.0309619*
Marsh-Flood Pool	3.135639	0.2 865401	5.984738	0.0257472*
River Fringe-Flood Pool	1.744967	−1.5448890	5.034823	0.4 994195
River Fringe-Marsh	−1.390672	−4.2397710	1.458427	0.5 690141

As a further insight in the microbial composition of these different samples, a compositional plot was drawn at the order level, where all taxa accounting for less than 2% of the relative abundance per sample were grouped as “Others” (Fig. 1C and D). To allow for an easy comparison between the two different sets of samples, only SML samples of the four breeding site types present in the larval dataset were included. In Fig. 1C, many different samples belonging to the same group have different compositional profiles, and a pattern cannot be clearly identified. In contrast, when looking at larval samples in Fig. 1D the profiles appear to be distinct in the different types of breeding sites, notable differences include fewer Enterobacterales in “construction site”, that overall appears to be completely different from the other breeding sites, with Burkholderiales dominating over other orders. Presence of Enterobacterales is reduced also in the “flood pool” group, which according to what can be observed in the PCA plot (Fig. 1B), is very similar in composition to the “river fringe” group; presence of Xanthomonadales is also characteristic of both groups. However, despite these similarities it can also be noted that Xanthomonadales seems to be frequently associated with Micrococcales in the “flood pool” group, while in the “river fringe” group the Micrococcales order is not prevalent, and what is more obvious is the presence of Cyanobacteria. Regarding samples from the "marsh" group, a higher presence of Enterobacterales can be recognized, along with the presence of Flavobacterales in many samples of the same location, suggesting the presence in some cases of more location-specific profiles.

To exclude the possibility that the observed differences in microbiota composition might be an artefact caused by drastically different complexity of the microbial community, alpha-diversity was analyzed using both observed species, and two of the most commonly utilized diversity indices, Shannon and Simpson index (Supplementary Fig. 4). Even with the use of different indices, the median of the two groups is always comparable, and the difference between groups is not significant according to a Kruskal–Wallis test (Observed: χ^2^ = 0.290, *P* = .58; Simpson: χ^2^ = 0.232, *P* = .63; Shannon: χ^2^ = 0.773, *P* = .37). The presence of more outliers shown from the broader range min-Q1 and Q3-max in the SML group highlight once again the high variability that characterizes the water samples. The result suggests that the complexity of the samples is not significantly different, thus the differences observed in the ordinations are real-life differences.

Moreover, since the compositional profiles of the larval samples (Fig. 1D) showed the presence of some location-specific patterns, we decided to explore how the site information is related to the type of breeding site. A new ordination was produced with labels corresponding to the location of collection (Supplementary Fig. 5). Furthermore, in Supplementary Table 5 a distance matrix between locations is provided. In three out of the four types of breeding site (since “construction site” is described from one single location) samples belonging to distant sites are still clustering together. Two sites (T, Q) belong to the “flood pool” type, 7051 m apart. Four sites belong to “marsh” type (Z, N, D, K) with distances ranging from 404 m (D, K) to 14 220 m (Z, K). Two locations belong to the “river fringe” group (B, W) with 23 461 m between them. Moreover, the one location of “construction site” type (location I) is 1709 m from location Z. This result suggests that there is no correlation between the closeness of the locations and their clustering in the PCA plot, which is instead determined from the type of breeding site alone.

All the previous results suggested that the microbial composition of a sample depends on the type of breeding site where it has been collected. However, we decided to test the observed effect further with the aid of a random forest (RF) classification model, with two main aims. The first aim was to assess how well the microbial data perform in classifying the breeding site type of the sample when using the microbial community data. Because our PERMANOVA results showed to be unreliable, the RF approach might be more suitable to the unbalanced nature of our dataset in which the groups do have different measurable spread, thus an accurate classification and subsequent permutational testing of the model performance can allow us to validate our conclusions on the observed distinction between breeding site types.

**Figure 2.**
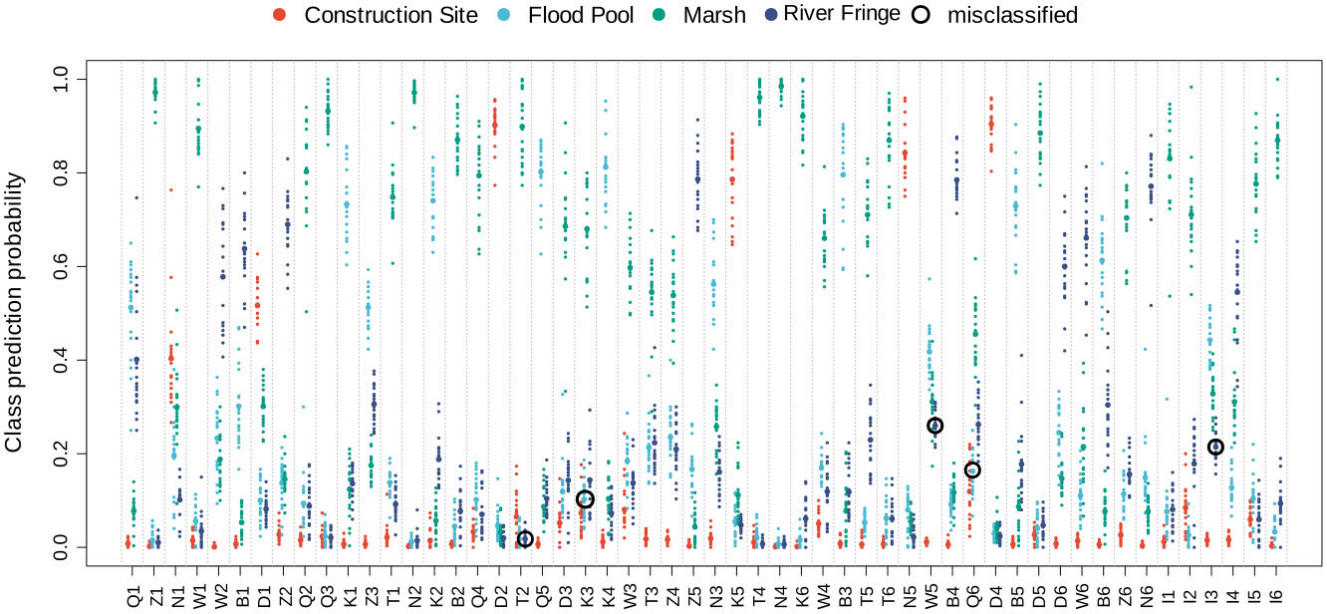
Swim lane plot for the results of the RF classification done on the larval dataset (MUVR repetitions: 20). Each lane corresponds to a sample, and for each repetition of the model, the class prediction probability for that sample (totalling 1 per repetition) gets registered among the four different classes. The individual repetition attempt results are represented by the small dots, while the average of all the repetitions is represented by a larger dot for each class. The higher the class prediction probability of a group, the larger the chance that that sample would be classified as belonging to it. Misclassifications are indicated by black circles, drawn around the correct class of a sample that has been classified mostly as a different one (higher prediction probability for an erroneous class). The colors of the different classes correspond to the type of breeding sites as in Fig. 1.

**Figure 3.**
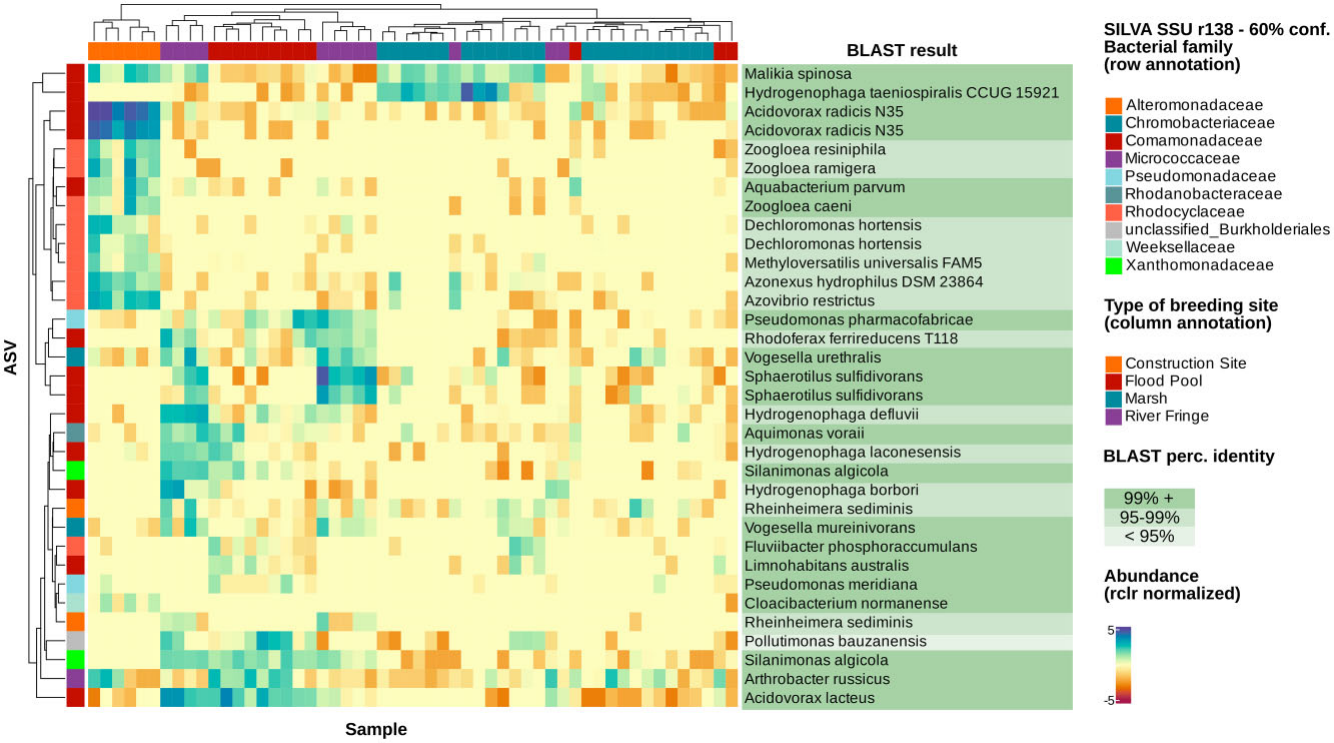
Heatmap of the abundance of the 34 ASVs selected by the RF classification model distinguishing between type of breeding site, from the model observed in Fig. 2. The values range between −5 (low abundance) to +5 (high abundance), the midpoint is zero and represents an actual abundance of 0. The samples and the ASVs have been clustered with a hierarchical clustering based on Euclidean distance, to highlight groups of ASVs that characterize a specific breeding site type. Furthermore, the heatmap has been complemented by species names obtained by running BLASTn for the ASV sequences, to facilitate the interpretation of clusters. This annotation has been highlighted with the percentage of identity to the top BLAST hit.

The classification model trained on larval data has a high performance with an accuracy of 91% (five misclassifications, *n* = 54) (Fig. 2); a permutational analysis of this model has also been computed (Supplementary Fig. 6), which reported a significant result (*P* = 8.046e−13). The result of the permutational analysis supports that the model built on larval microbial abundance can classify the group to which a sample belongs to with high accuracy compared to a population of randomly assigned group flags, thus proving that the microbial community between different types of breeding sites is different. The same kind of model trained on SML data (Supplementary Fig. 7) shows a very low accuracy in the classification, with multiple misclassifications exceeding 50% (the model has been trained only on the four classes present on the larval dataset, to allow direct comparison). A permutational analysis has been computed also for the SML model (Supplementary Fig. 8) (*P* = .09157), which shows how the SML microbial composition is not able to distinguish between different types of breeding sites, suggesting a higher breeding-site specificity in larvae than in water. The second aim was to use the rdCV scheme implemented in the algorithm to select important taxa that are driving the classification. ASVs were extracted from the mid-conservative model (see results for an explanation) (*n* = 34) and their abundance was analyzed through a heatmap (Fig. 3). BLAST search complemented our existing taxonomical classification to further explain the single identified ASVs.

### Overlap between surface microlayer and larval microbiota

The larval microbiota composition is heavily influenced by the microbial community at the breeding site. However, to understand why there is such a strong association between the microbiota of larvae and the type of breeding site, while this association is much weaker for the SML, we decided to explore how these two communities overlap and if the species that are incorporated by the larvae differ according to type of breeding site.

For this analysis, only SML samples from locations with mosquitoes were included.

**Figure 4.**
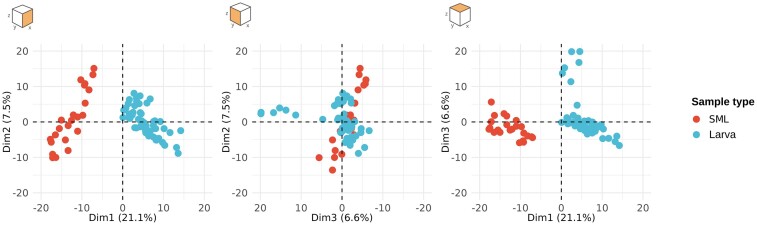
PCA including both larval and SML dataset. The difference in composition is mainly expressed along the first dimension of the data, explaining 21.1% of the total variance.

A PCA (Fig. 4) shows that the larval and SML samples are clearly distinct across multiple axes (PERMANOVA: *R*^2^ = 0.19, *P* = .001), with the first three PCs accounting approximately for 35% of the total variance. However, when testing for homogeneity of multivariate dispersion, it resulted in a significantly different dispersion (*F* = 28.94, *P* = .001). So, to confirm the degree of dissimilarity we used again an RF classification approach, which resulted in 100% accuracy with a very low number of variables (Supplementary Fig. 9), indicating clearly distinct microbial profiles for the two different groups and confirming our observations based on the ordination. Then, compositional profiles were also drawn (Supplementary Fig. 10), where the different taxa present in the samples are presented on the order level. The profiles of SML and larval microbiota appear to be rather different, at least for the species accounting for most of the abundance. The presence of Burkholderiales is higher in larval samples than in SML samples, Enterobacterales are almost exclusively associated with larval samples. In particular the family *Thorselliaceae*, of which two genera are present (*Thorsellia* and *Coetzeea*) is present in all the larval samples, with a total of 49 589 counts among 20 ASVs, while in the SML samples the whole family had a total count of 2 (Supplementary Table 6). The exact opposite behavior can be observed for Pseudomonadales, which are much abundant in the SML samples, together with Flavobacteriales. Both the PCA and the profiles highlight how the overlap between the two datasets is relatively small.

**Figure 5.**
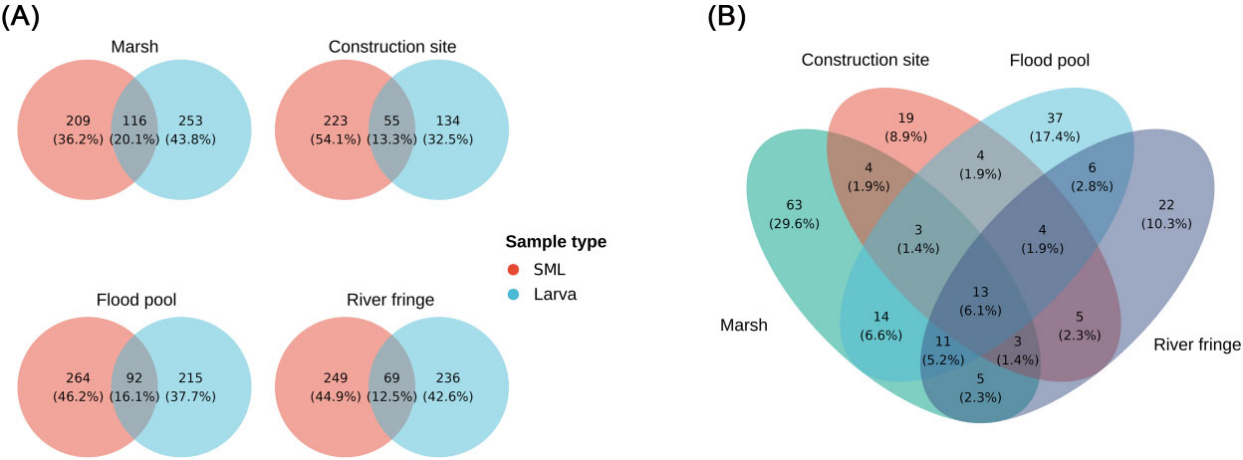
(A) Number of shared ASVs between SML and larval dataset, for each type of breeding site. The percentage of shared members is in a small range, with the lowest one being the share between the two datasets from the “River fringe” type and the highest from samples belonging to the “Marsh” type. (B) An overlap of the shared species for each type is represented, suggesting a relatively low overlap, or the absence of a consistent incorporation across different types of breeding sites.

Regarding shared ASVs, the total number of ASVs in the SML samples is 540, while for the larval samples that amounts to 444. Of those ASVs, 36.6% is shared, totalling 262 shared ASVs. The proportion of shared ASVs is at the same level when separating the data according to breeding site. For all four breeding site types the percentage of ASVs that gets incorporated by larvae is oscillating in the range of 12.5–20.1% (Fig. 5A), suggesting that this low incorporation happens all through the dataset. Moreover, the difference in number of bacteria that are incorporated across different breeding site types, although being very small, can be an indication of more ASVs suitable for survival in the larval gut being present in one type rather than another. We considered whether the differences in the incorporated number of ASVs might depend on different microbiota complexity at different sites. Testing the significance of the difference in alpha diversity between the different types of breeding sites did not yield significant results when tested on Shannon index with a Kruskal–Wallis test (χ^2^ = 10.941, *P*-value = .36); hence, this difference can be explained by the actual composition rather than from the differences in complexity of the microbial communities.

**Figure 6.**
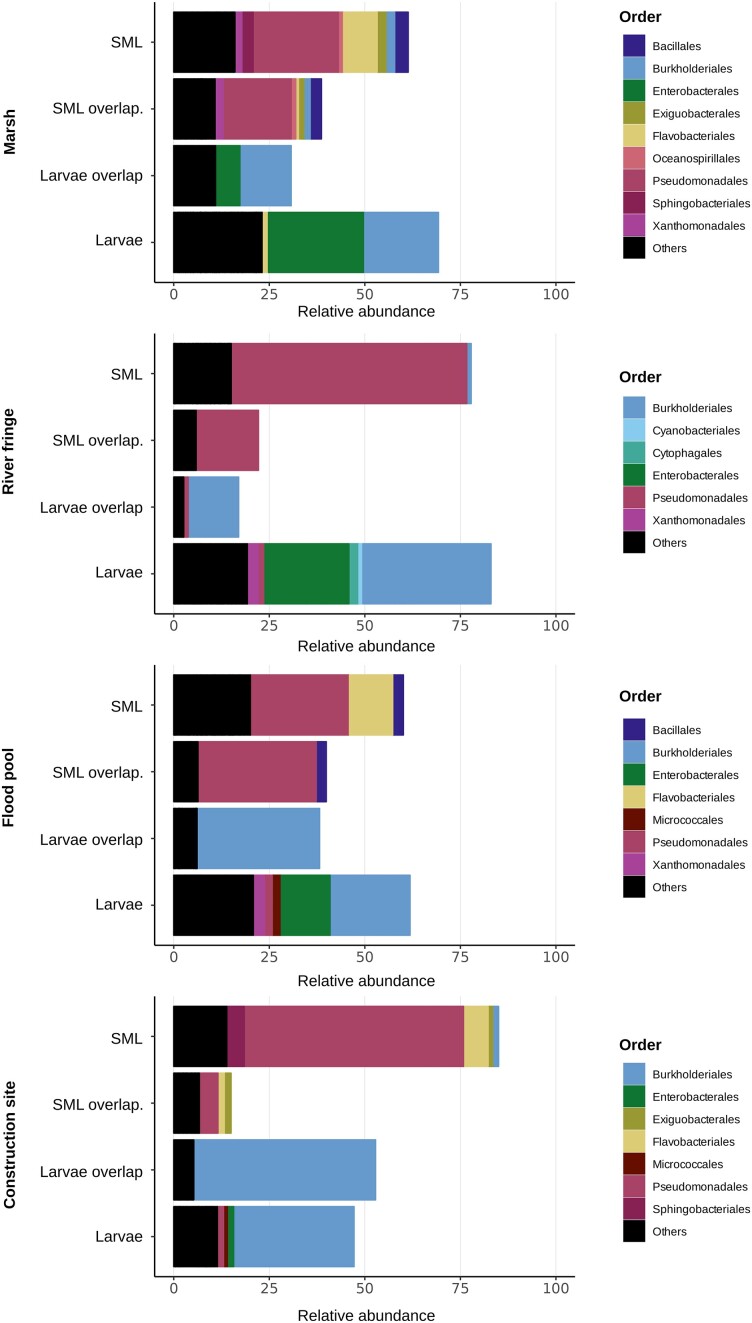
Relative abundance of bacterial orders divided across the four different types of breeding sites considered in the study. In each type of breeding site, the relative abundance was calculated separately for SML and larval datasets, and the portion corresponding to the overlapping ASVs is represented in a distinct group, to highlight the change in relative abundance of these ASVs between the two sets of data. Despite being constituted by the same bacteria, the two “overlap” groups are drastically different from each other.

The overlap of the different ASVs obtained from larvae in the four different types of breeding sites is shown in (Fig. 5B). While the overlap suggests large differences in terms of ASVs across the different types of breeding sites, we also wanted to compare the composition on a higher taxonomic level. The composition of these overlaps (Fig. 6) highlights that larval samples always contain Burkholderiales and Enterobacterales independent of the type of breeding site. The plot in Fig. 6 also shows the difference in relative percentages of the ASVs that constitute the overlaps. In all the different breeding sites the overlap reflects the original water composition; most of the orders that are present in the water are also part of the overlap, despite the drastic change in relative abundance from water to larval environment. Moreover, the portion of the total microbiota that is part of the overlap is quite different among different types of breeding sites, with “construction site” being the highest with approximately 50% of the total larval microbiota being constituted by overlap bacteria, while the lowest being “river fringe” with approximately 20%.

### Water physico-chemical parameters influence the presence indicator species

As described in the section "Collection of surface-micro layer and larval samples", some locations in which the collection took place did not yield any larval samples. Since oviposition can be driven by volatile organic compounds (VOCs) and other chemical or physical features, influenced by the microbiota composition in a specific location (Girard et al. 2021), we hypothesized that the SML belonging to the water/air interface, might play a key role in this. Since SML microbiota composition in water samples might be drastically influenced by the different physico-chemical parameters in each location, we decided to explore their relation to SML microbiota and presence/absence of mosquitoes.

We made use of an indicator species analysis (ISA) to retrieve the ASVs with a higher diagnostic power for both groups. In short, ISA produces an indicator value for each species, that is based on “specificity” and “sensitivity”, expressing respectively the probability that the surveyed site belongs to the target site group, and the probability of finding the species in sites belonging to the site group.

**Figure 7.**
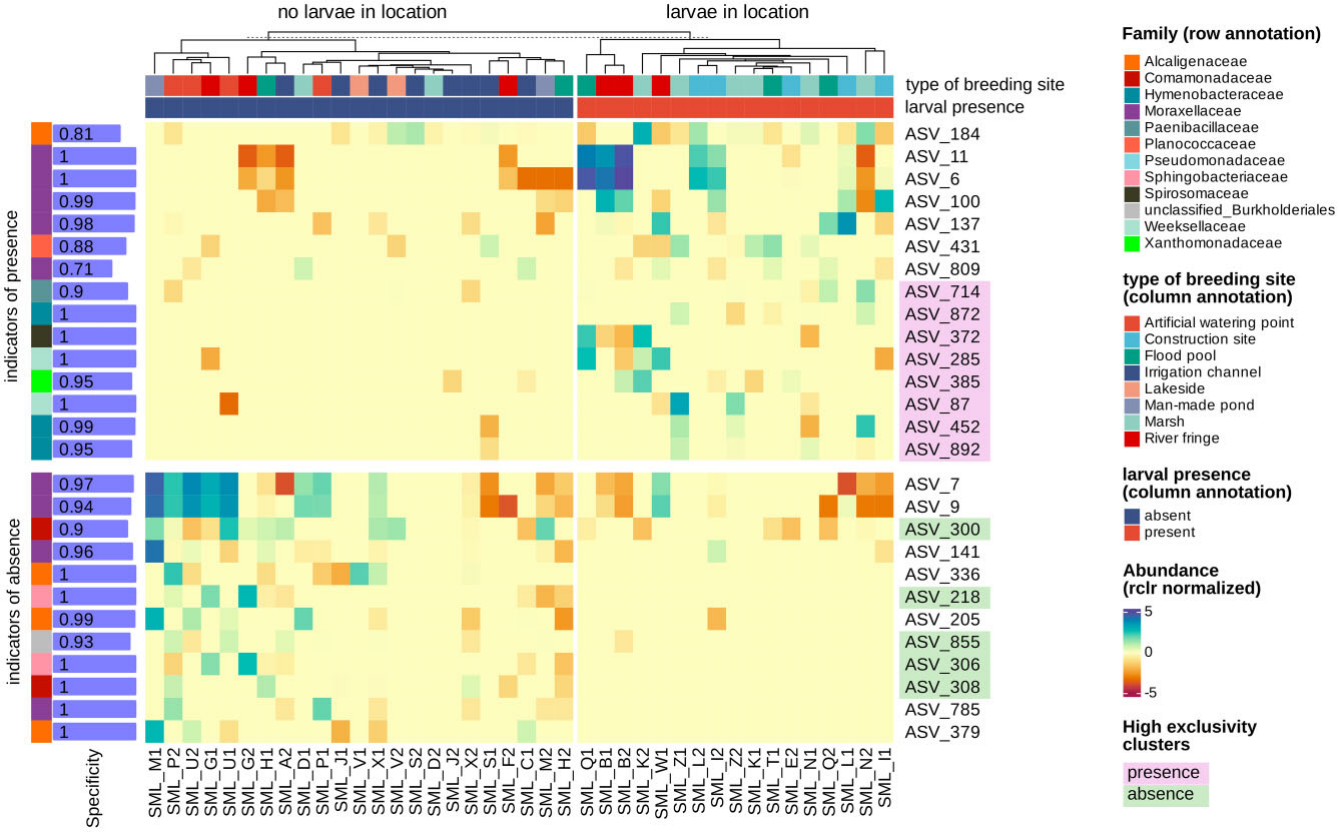
Heatmap of the normalized abundance of indicator species of larval presence or absence (ASVs, *P*-value < .05). The specificity of each resulting ASVs is indicated on the left (range 0–1), it is possible to identify multiple ASVs that have an extremely high specificity and are also exclusive families of either of the two conditions (presence or absence), these are highlighted on the right of the heatmap. Samples have been divided prior hierarchical clustering into the two groups indicated at the top, based on larval presence at collection.

ASVs reported in Fig. 7 were significant (*P* < .05), having a positive predictive value, expressing the likelihood that a site will harbour mosquito larvae according to the presence of the specified taxa. Among those ASVs, there are some belonging to families that are exclusive to each group. From the ASVs indicators of presence of larvae, a cluster constituted by *Paenibacillaceae, Hymenobacteraceae​, Spirosomaceae​, Weeksellaceae​*, and *Xanthomonadaceae*​ can be identified. This is opposed to a cluster of *Comamonadaceae​, Sphingobacteriaceae​*, and *Bulkholderiales* that are instead indicators of absence.

*Weeksellaceae​* and *Spirosomaceae* (phylum Bacteroidota) always had a specificity of 1, indicating that in 100% of the instances in which their ASVs were found, the location harboured mosquito larvae, suggesting a possible involvement in mosquito attraction. Regarding the indicator of absence, they surprisingly include an unclassified species of *Bulkholderiales*, one of the main orders present in larval microbiota (see Fig. 6), however with a slightly lower specificity, indicating its presence also in locations where mosquitoes were found. *Sphingobacteriaceae* appears to be present with a specificity of 1, as a strong indicator of absence of mosquitoes, along with two ASVs of *Comamonadaceae*.

**Figure 8.**
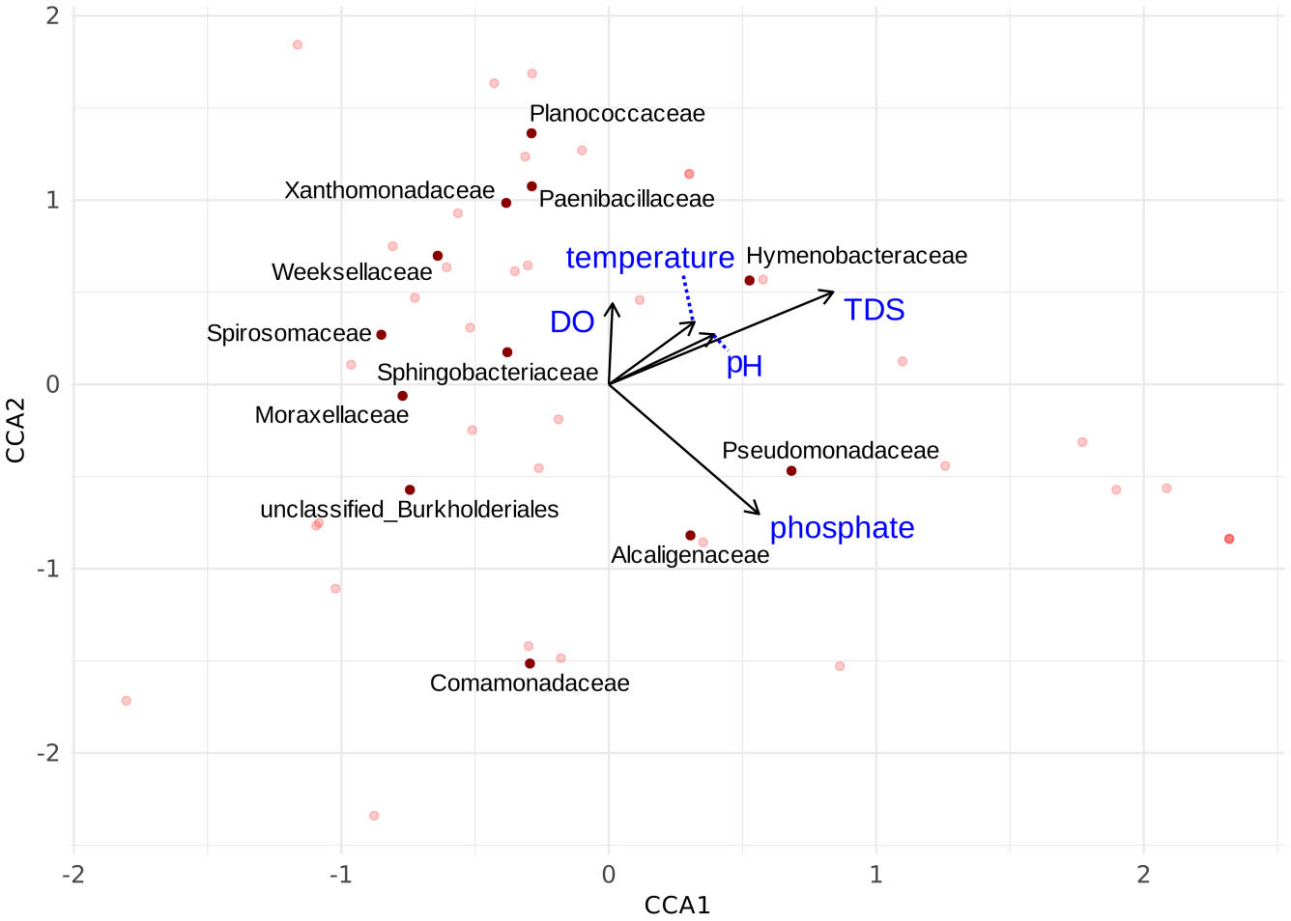
CCA showing families selected as indicator species and their relationship with environmental variables constraining the analysis. The relation's strength is proportional to the arrow's length and the direction reflects the sign of the correlation, either positive or negative (other bacterial families have been used to produce the ordination but are shown with a lighter color in the plot).

The results of this ISA are accompanied by a CCA performed on the SML microbiota (Fig. 8). Physico-chemical parameters to include were selected according to the use of a cross-correlation matrix (Supplementary Fig. 11), from which it appears that "total dissolved solids" (TDS) and “electrical conductivity” (EC) are almost exact covariates, “turbidity” and “phosphate” also presented a very similar cross-correlation. Variables less expected to influence microbial composition as “turbidity” and EC were removed from the constrained analysis. It is also worth noting that “turbidity” and “phosphates” did not include replicates, because the sampling method itself does inadvertently alter the site properties. These two measurements might then not be as reliable as the other physico-chemical parameters.

Highlighted families are the ones that have been selected as indicators either for presence or absence. Regarding the indicators of presence, *Hymenobacteraceae* positively correlates with TDS, while *Spirosomaceae, Weeksellaceae​*, and *Xanthomonadaceae*​, all show an inverse correlation with Phosphate. For the indicators of absence, *Comamonadaceae* and *unclassified_Burkholderiales* both negatively correlate with TDS, while *Comamonadaceae* also exhibits a slightly positive correlation with Phosphate​, suggesting that changes in physico-chemical indicators, this might influence the suitability of a location to oviposition even through promoting/inhibiting some microbial species.

## Discussion

Based on our results, it appears that the environmental characteristics of a breeding site (we refer to it as “type” in the context of this work) may influence the composition of the surface microlayer (SML) to a smaller degree than the composition of larval microbiota. A speculation on this effect is that it might be depending on the interaction between different inputs in different types of sites, and the larval microbiota. Some examples of components influencing mosquito detectability have been previously identified in literature as proximity to vegetation, or to running water (Low et al. 2016). These, and others, might be characteristics which differ between different sites, and determine this kind of effects.

Moreover, the effect of the surrounding environment on the larval microbiota probably accumulates over time and changes at a slower pace than the surrounding water microbiota.

The larval samples from the 16 selected sites clustered according to which type of environment they belong to, and the composition of the mosquito microbiota is less diverse than the SML microbiota at the corresponding sampling site, as shown in Fig. 1. Different species of bacteria are colonizing the breeding sites, creating different ecological niches (Odling-Smee et al. 2013). However, the reason why the association to a certain breeding site type is more pronounced in larval samples, than in water, may lie in the interaction between the mosquito gut environment and the organic matter and bacterial population in the location where they are feeding. Microbial communities secrete exopolymers and dissolved organic matter, some of which are easily digestible, contributing together to the environment in the mosquito gut (Wotton et al. 1997). The combination of secondary metabolites, different lysates from bacterial substrates, and changes in this gut micro-environment may determine an accentuation of the selective effect of the water environment that is taking place in each location because of trans-generational transfer events such as egg-smearing (Favia et al. 2007) and trans-stadial transfer (Lindh et al. 2008). Concerning bacterial composition of the larval samples, the families identified here correspond to what has been observed also in *Anopheles gambiae* mosquitoes, in which the presence of beta and gamma proteobacteria constitutes a large proportion of total abundance (Gimonneau et al. 2014, Buck et al. 2016).

We employed RF modelling to identify taxa driving the difference in larval microbiome across different types of breeding sites (Fig. 3). Using this approach, we identified the high abundance of *Rhodocyclaceae* and *Comamonadaceae* to be particularly important in clustering larvae from artificial locations (construction site) apart from the other groups. In particular, *Comamonadaceae* have been observed to be present in wastewater treatment plants (Wang et al. 2020), which might explain their higher abundance in the “construction site” location due to the possible presence of substrates of human origin. Moreover, among them there are members of the genus *Zooglea*, a group of floc-forming bacteria, which is interesting because their contribution to the creation of biofilm could be the reason this genus is retained easily in mosquito larvae when present in the water. Another cluster including *Comamonadaceae, Chromobacteraceae* but also *Xanthomonadaceae* seems to be particularly important in “river fringe” and “flood pool” samples, with a clear reduction of *Sphaerotilus* and *Vogesella* in “flood pool”. However, it can be seen how also in this case it is possible to identify ASVs that are able to distinguish between the two. Misclassifications in the model are samples B2, T2, and T5 (Fig. 2), the first from the “river fringe” group and the last two from the “flood pool” group, all classified erroneously as marshes, a confusion that might be generated by a partial overlap between these groups, because the “marsh” group is also the one including more samples, which might drive this trend. Also, from the misclassification of W4 and W6 from the “river fringe”, identified as flood pools, it appears that the microbiota in these two groups is at least partially similar, which suggests a similar kind of micro-environment. Altogether these results suggest that bacterial communities in larvae are indeed influenced by the type of breeding site.

Moreover, considering the discrepancy between outcomes of the two classification models on larval samples and SML (Fig. 2 and Supplementary Fig. 7), the lack of association between breeding site and SML microbiota composition suggests that microbiota composition in surface water is driven predominantly by other factors or is subject to large random variability. This, in combination with the strong association between breeding site type and microbiota composition in the larvae, further suggests that their microbiota composition is seemingly driven by other factors. These results are largely in line with the lack of overlap between SML and larvae observed in the ordinations. Overall, these results suggest that the composition of microbiota in the larval micro-environment is much more tied to the type of breeding site, compared to its SML counterpart.

Then we further characterized this overlap between larval microbiota and the SML. As previously discussed, the development of mosquito microbiota is intertwined with the location in which larvae develop (Gimonneau et al. 2014). When considering the microbiota of the water without a specific focus on the SML, microbiota in larvae have been shown to be constituted by the same families present in the water, in multiple genera of mosquitoes (Coon et al. 2014), even if their relative abundances change considerably. In these studies, the total overlap between taxonomical units in water and larval samples has been shown to be around 34% (Coon et al. 2014). The percentage of species retained from the SML has been shown in other studies focusing on *A. gambiae* to be close to 59% (Gimonneau et al. 2014), suggesting that the gut environment of the larvae is selective towards specific species. In *A. arabiensis*, we found that SML and larvae do not share such a high percentage as previously shown, rather only 36.6% of the total number of taxonomical units is shared. Although this could be explained by the use of denoising rather than clustering algorithms, providing more resolution for the identification of rare or low abundance taxa, we find that the percentage of shared taxa, being lower than expected, is a good indication that the contribution of other selective mechanisms is playing a big role in shaping larval microbiota. For example, as shown by others (Lindh et al. 2008, Coon et al. 2014), trans-stadial transfer could be an important explanatory factor.

We hypothesized that bacteria that are actually being incorporated during larval development are species that would likely be more attuned to survive in a larval environment, thus contributing to some sort of “core microbiota” (Garros et al. 2008). The number of shared ASVs between the different sites is rather low, suggesting that mosquitoes are not selectively incorporating specific ASVs.

In addition to that, it can be noted that the taxa included in the overlap are almost mirroring completely the composition of the aquatic SML environment (Fig. 6), suggesting a rather indiscriminate incorporation of bacterial species. Interestingly, the ASVs that are incorporated in the larval microbiota, change their relative percentage drastically as it can be observed from the composition of the “overlap” groups (Fig. 6). This effect might reflect the magnitude of changes bacterial species go through because of adaptation to the internal environment of the mosquito (Garros et al. 2008).

However, there is one genus that has been identified in all breeding sites, *Thorsellia* (Supplementary Table. 6). It belongs to the family *Thorselliaceae* (Kämpfer et al. 2016) and is a genus of bacteria so far exclusively found in the gut of mosquito species known to be major vectors of disease (mainly malaria vectors). The first species, *Thorsellia anophelis*, was originally isolated in Kenya from *A. arabiensis* (Lindh et al. 2005, Kämpfer et al. 2006) and was the dominant genus in a Kenyan population of *A. gambiae* mosquitoes where 40% of the adults harboured *Thorsellia* (Briones et al. 2008). Another study found that almost 70% of the bacteria in young adults belonged to the *Thorsellia* genus (Wang et al. 2011). Analyses of the properties of *Thorsellia* showed that it has adapted to mosquito guts by accepting the alkaline pH found in mosquito larvae and growing faster in blood culture (Briones et al. 2008), which could explain its omnipresence.

Members of *Thorselliaceae* (*Coetzeea* and *Thorsellia*) have now been found in vectors of malaria in Africa (Akorli et al. 2016, Segata et al. 2016), Asia (Chavshin et al. 2014, Ngo et al. 2015) and South America (Kämpfer et al. 2016), but also in *Culex* mosquitoes from California (Duguma et al. 2015). According to BLAST results (Supplementary Table 6), ASVs representing *Thorsellia* in the present study belong mostly to *T. kenyensis*, with only two ASV representing *T. kandunguensis* and *Coetzeea brasiliensis*, respectively. It was also shown that *T. anophelis* was the most commonly occurring bacteria found in reproductive tissues of *A. gambiae* and *A. coluzzii*, which could indicate that it is vertically transmitted (Segata et al. 2016). The possibility of vertical transmission of *Thorsellia* is supported by the presence of *Thorsellia* in all the larval samples of this study, but almost complete lack of *Thorsellia* in their breeding waters.

Furthermore, we investigated the link between physico-chemical parameters and water microbiota, and how these correlate to larval presence. Interactions between physico-chemical parameters and mosquito presence have been studied before, examples include *A. gambiae* in Kenya (Mala and Irungu 2011), *Anopheles* larvae in Ethiopia (Low et al. 2016), and *Aedes aegypti* in Burkina Faso (Ouédraogo et al. 2022). Less common are studies on the connection between microbiota or bacterial-induced chemical cues and larval presence/absence, for example a study concerning *Ae. aegypti* (Ponnusamy et al. 2008) and a study from Onchuru et al. (2016) on *Aedes, Anopheles*, and *Culex* mosquitoes in Kenya.

In our study, we aimed to describe not only the microbial communities associated specifically with the *Anopheles* genus but also identified indicator species in the SML microbiota for the presence of mosquito larvae. Then, we correlated those species with relevant physico-chemical parameters, following the hypothesis that physico-chemical characteristics of the water can influence oviposition indirectly, through their effect on microbial profiles and the volatiles that microbial species produce, which can have an attractive effect on egg-laying mosquitoes (Lindh et al. 2008, Girard et al. 2021).

We found that *Hymenobacteraceae* was an indicator of larval presence and had a strong positive correlation to TDS (Fig. 7). Previously, it has been shown that a higher concentration of TDS might be important for the development of larvae in locations populated by anopheline larvae (Mala and Irungu 2011). Likewise, a study done in *Aedes* larvae showed how TDS is positively associated with larval density and how EC (a function of TDS) positively correlates with an increase in body site (Ouédraogo et al. 2022). Among the other families affected by TDS, we found also *Comamonadaceae* and *Burkholderiales*, which in our results were classified as indicators of larval absence and were instead negatively correlated to the TDS concentration. At the same time, our results also show *Spirosomaceae, Weeksellaceae*, and *Xanthomonadaceae* as indicators of presence being negatively correlated with phosphate, while *Comamonadaceae* (an indicator of absence) has a positive correlation with phosphate. Phosphates have been shown before to correlate positively with *Aedes* presence while *Anopheles* mosquitoes were not affected by their concentration (Onchuru et al. 2016). In our results we support that despite this absence of direct correlation, there is still a negative influence of phosphates on bacterial species that seem to be preferred by *A. arabiensis* larvae, while bacterial species that are indicators of absence increase with an increase in phosphates (*Comamonadaceae*). Moreover, while phosphates were not directly assessed in a study concerning *Anopheles* mosquitoes (Low et al. 2016), the authors did focus on riparian vegetation, proving that an increase in vegetation coverage could reduce the presence of larvae. Since sources of phosphates in water include decaying plant matter, as well as fertilizers, their presence might be directly correlated to riparian vegetation, suggesting that an increase in vegetation coverage, and thus phosphate concentration, might boost the development of bacterial species that seem to be unattractive to *Anopheles* mosquitoes. Results such as these emphasize the complexity of the interactions between mosquitoes and their environment. Riparian vegetation can act as a physical barrier to ovipositing female mosquitoes, may influence the flow of nutrient by capturing compounds moving through the soil and may further influence the water by compounds released by plant material falling in the water.

Previous studies found differences in mosquito microbiota across locations, for instance in a study focusing on water containers in settings with anthropic activity done on *Aedes* mosquitoes in Puerto Rico (Caragata et al. 2022), a significant difference in the number of families was detected between container types, proving that the nature of the breeding site is indeed an influencing factor that shapes microbial communities in mosquitoes. In *Anopheles* mosquitoes it has been observed that when emerging from different aquatic habitats, individuals harbour different bacterial species (Buck et al. 2016), and while the inter-individual variability drops from the larval to the adult stage, the inter-locality variability carries over from the larvae to the adult (Gimonneau et al. 2014). Similarly, another study in Ghana highlighted the difference in microbial composition between rural and urban settings in *Anopheles* mosquitoes, in both diversity and taxonomic composition (Akorli et al. 2016).

In conclusion, the classification of breeding sites in clear categories according to the surrounding environment allowed us to expand on previous work and further characterize the microbiota of *A. arabiensis* vector mosquitoes. Moreover, the use of machine learning methods allowed for identification of important features that might drive separation between different types of breeding sites. We also characterized the overlap between larval and water microbiota and found novel results compared to what has been previously observed and showed how much the relative abundance of the incorporated bacterial species changes between the two despite the intimate relationship between mosquito larvae and the water bodies they inhabit. Lastly, on physico-chemical parameters, our results suggest that variations might influence mosquito development through the influence on important bacterial families. These families might even be involved in the release of compounds related to mosquito attractiveness and oviposition (Ponnusamy et al. 2008, Girard et al. 2021). While the results of this study may be limited in scope, they have the potential to be a starting point for further developing our knowledge of *A. arabiensis* mosquitoes and their interaction with the SML environment. Therefore, it will be important for the ideas presented here to undergo experimental testing to validate their robustness and reproducibility.

## Supplementary Material

fiae161_Supplemental_Files

## Data Availability

The raw Ilumina paired-end reads used in this analysis are available on the ENA under the accession number: PRJEB76915.
