# Supplementary material for: The type of environment has a greater impact on the larval microbiota of Anopheles arabiensis than on the microbiota of their breeding water
Source: FEMS Microbiol Ecol. 2024 Dec 18;101(1):fiae161. doi: 10.1093/femsec/fiae161 (PMC11737318; doi:10.1093/femsec/fiae161)
Supplement: fiae161_Supplemental_Files [file fiae161_supplemental_files.zip › supplementary_data_captions.docx]

**Figures**

**Supplementary Figure 1 -** A) map of the region where the sampling was collected, along with the location of the 26 collection sites. In fig B-E, examples of the breeding site types included in the study; construction site (B), river fringe (C), marsh (D) and flood pool (E).

**Supplementary Figure 2 -** Bar plot illustrating the number of samples for each breeding site type, in the larval (top) and SML (bottom) dataset.

**Supplementary Figure 3 -** Overview of the result of the rarefaction/extrapolation procedure from iNEXT on the two different datasets, larvae (left column) and SML (right column) on two different alpha diversity indices. The plots show however an increase in observed species with increasing sample size (number of reads). When taking into account Shannon diversity, both datasets reach a plateau quite early, indicating an accurate representation of the complexity of each sample.

**Supplementary Figure 4 -** Boxplot representing the alpha diversity of the two sample groups (SML and mosquito samples). The plots refer to different diversity indices, from left to right: Observed species, Shannon index and Simpson diversity index, which all show a comparable median between the two groups.

**Supplementary Figure 5 -** Principal component analysis on the larval dataset. The shape of the points corresponds to the location where the samples have been collected, while the color indicates the type of breeding site for each location. Samples belonging to locations which are closer to each other cluster according to the type of site.

**Supplementary Figure 6 -** Permutational analysis on the classification model trained on the larval dataset. The accuracy of the model and its number of misclassifications are compared with a population of models built with random label assignment, which has a misclassification average of roughly 2/3 of the total dataset size. The further our trained model places itself from the random population, the more significant its result.

**Supplementary Figure 7 -** Swim lane plot of the classification model trained on water samples.

**Supplementary Figure 8 -** Permutational analysis on the classification model trained on the SML dataset. Compared to the permutational analysis in Supplementary Fig. 6, it can be noted how, when using SML data, the performance is comparable to the random models population. This suggests a low impact of SML microbiota composition in classifying the type of breeding site.

**Supplementary figure 9 -** result of the RF model to classify between SML and larval dataset (A), and permutational analysis of the model (B). The result shows 0 misclassifications, with superb accuracy in discerning the two kinds of samples.

**Supplementary Figure 10 -** Compositional profile comparing relative abundance of bacterial orders in the larval and the SML dataset. The composition appears very different, and shared species have different relative abundances. All the ASVs with a relative abundance of less than 2% of the sample total abundance were grouped in as "Others".

**Supplementary Figure 11 -** Heatmap of cross-correlation between environmental variables and the families in the SML dataset. Highly correlated pairs are to be expected to have a positive correlation with each other, and vice-versa.

**Tables**

**Supplementary table 1 -** Table containing an overview of the larval and SML samples' collection location.

**Supplementary table 2 -** Table containing an evaluation of DADA2 performance, samples are in the rows. Each column reports the number of reads passing that specific step, the final percentage of reads retained is reported at the end. Column “filtered” reports the result of DADA2 filtering procedure, “DADA_f” and “DADA_r” report the result of the denoising algorithm for forward and reverse reads, “merged” reports the number of successfully merged reads, “non_chimeric” reports the number of sequences flagged as non_chimeric and retained in the end.

**Supplementary table 3 -** Table reporting an evaluation of taxonomic classification from DECIPHER. The number of unclassified ASVs is reported at each taxonomic level, along with the number of “NA” (an unclassified sequence at a specific taxonomic level is reported as NA in the successive ones). Also, the “tot. uncertain” reports the sum of NA and unclassified, the “identified” reports the total number of successfully classified ASVs, and “taxa no.” reports the number of unique taxa identified at each level. Finally, the “Percentage” column reports the percentage of non-identified ASVs at each level.

**Supplementary table 4 -** Table reporting coverage analysis per sample according to the iNEXT package. Columns description: reference sample size (Reads), observed species richness (S.obs), a sample coverage estimate (SC), and the first ten incidence frequency counts (f1-f10)

**Supplementary table 5 -** Distances between different villages calculated according to GPS coordinates. Distances expressed in meters (m).

**Supplementary table 6 -** ASV table of family *Thorselliaceae*. Total counts for each ASVs can be found for water and larval samples, and the genus of each ASV is indicated.
