# Supplementary figures and images for: The type of environment has a greater impact on the larval microbiota of Anopheles arabiensis than on the microbiota of their breeding water
Source: FEMS Microbiol Ecol. 2024 Dec 18;101(1):fiae161. doi: 10.1093/femsec/fiae161 (PMC11737318; doi:10.1093/femsec/fiae161)

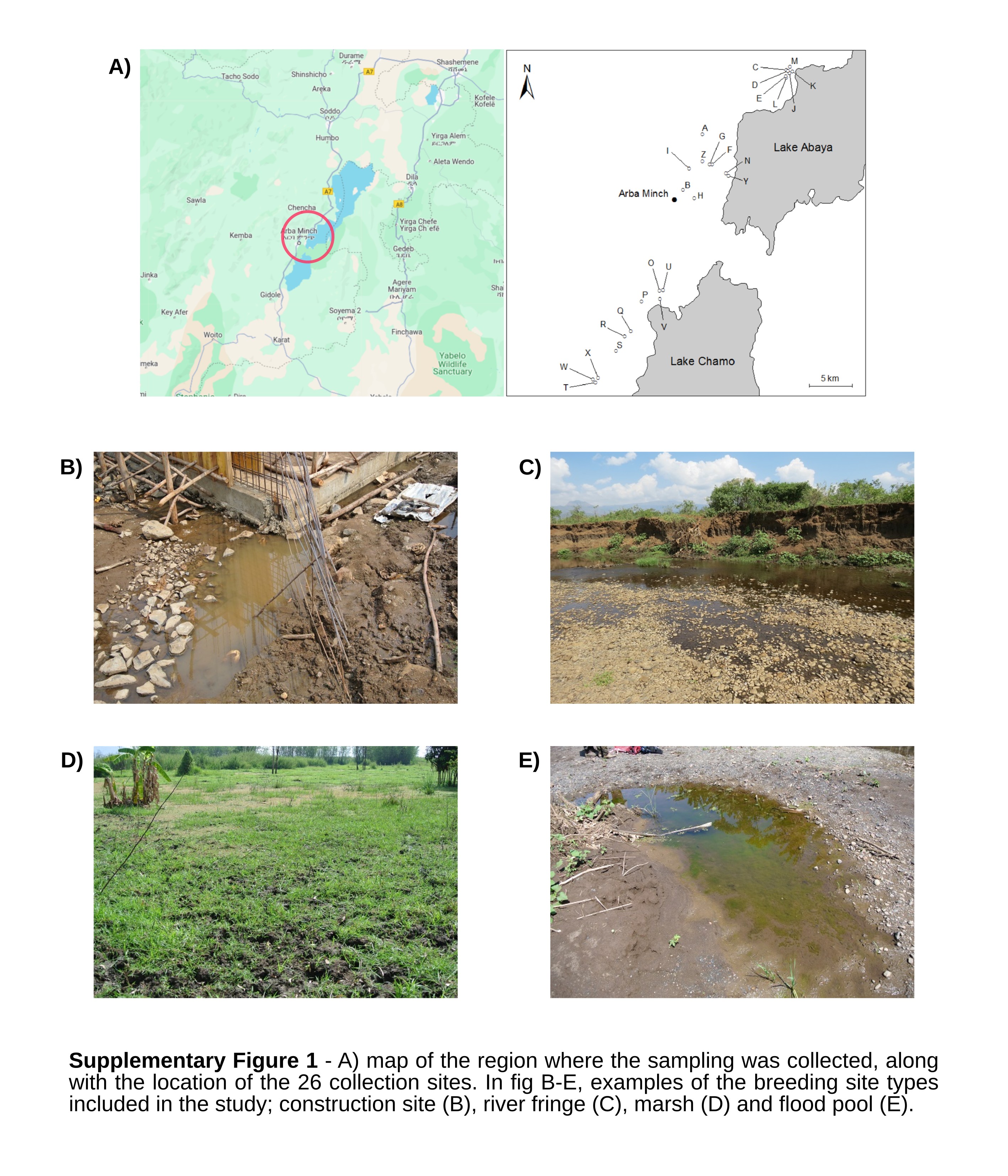

Supplement: fiae161_Supplemental_Files [file fiae161_supplemental_files.zip › supplementary_data-figure_1.jpg]

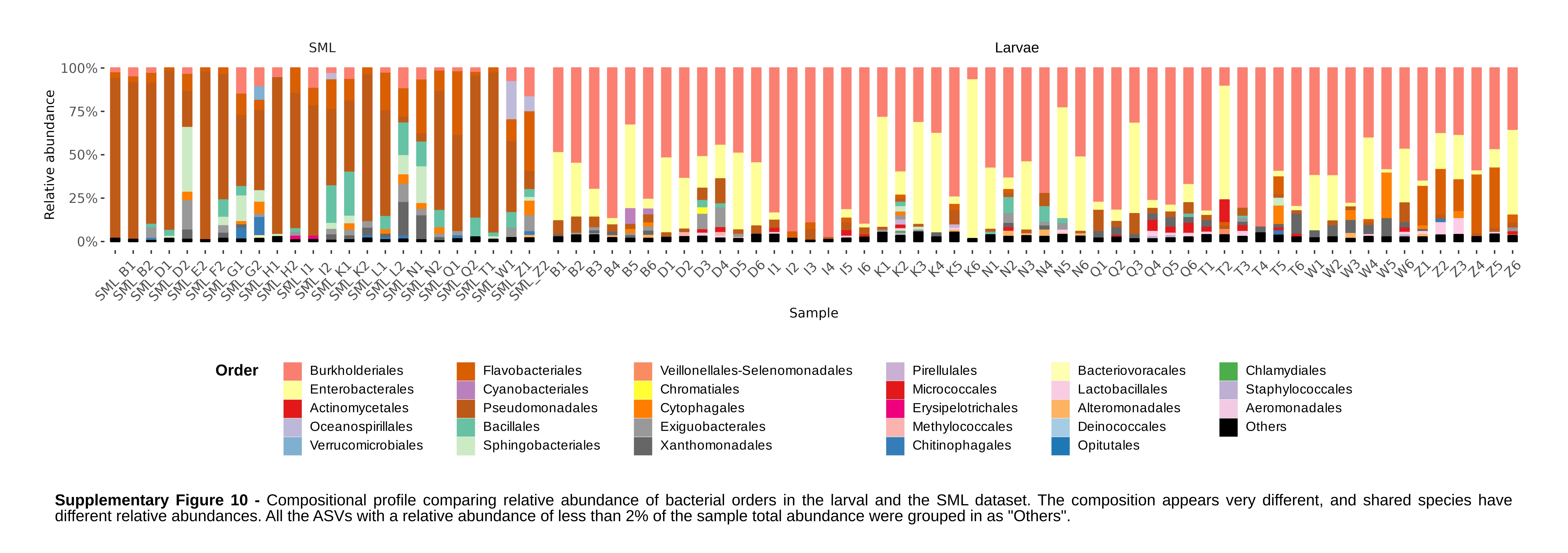

Supplement: fiae161_Supplemental_Files [file fiae161_supplemental_files.zip › supplementary_data-figure_10.jpg]

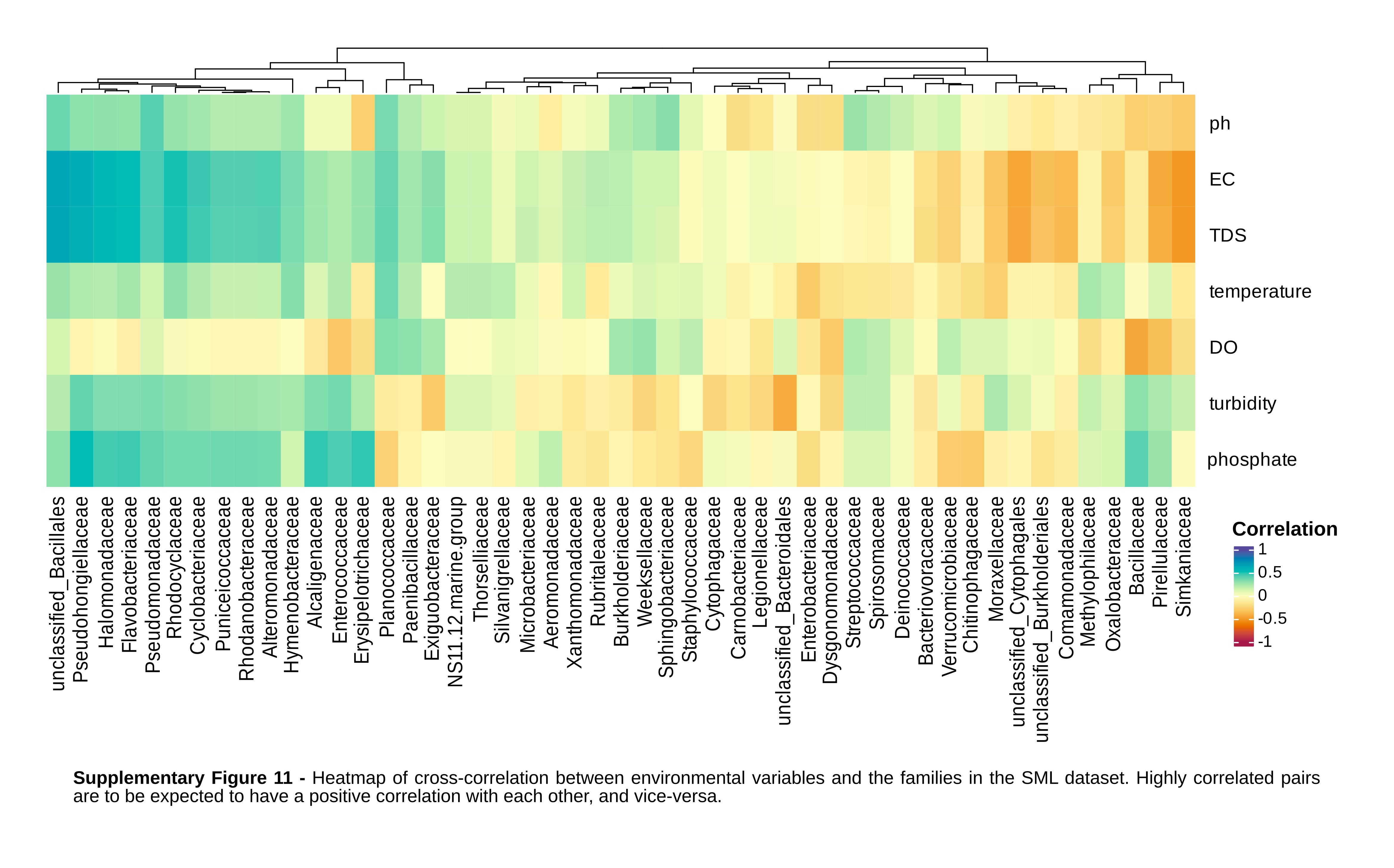

Supplement: fiae161_Supplemental_Files [file fiae161_supplemental_files.zip › supplementary_data-figure_11.jpg]

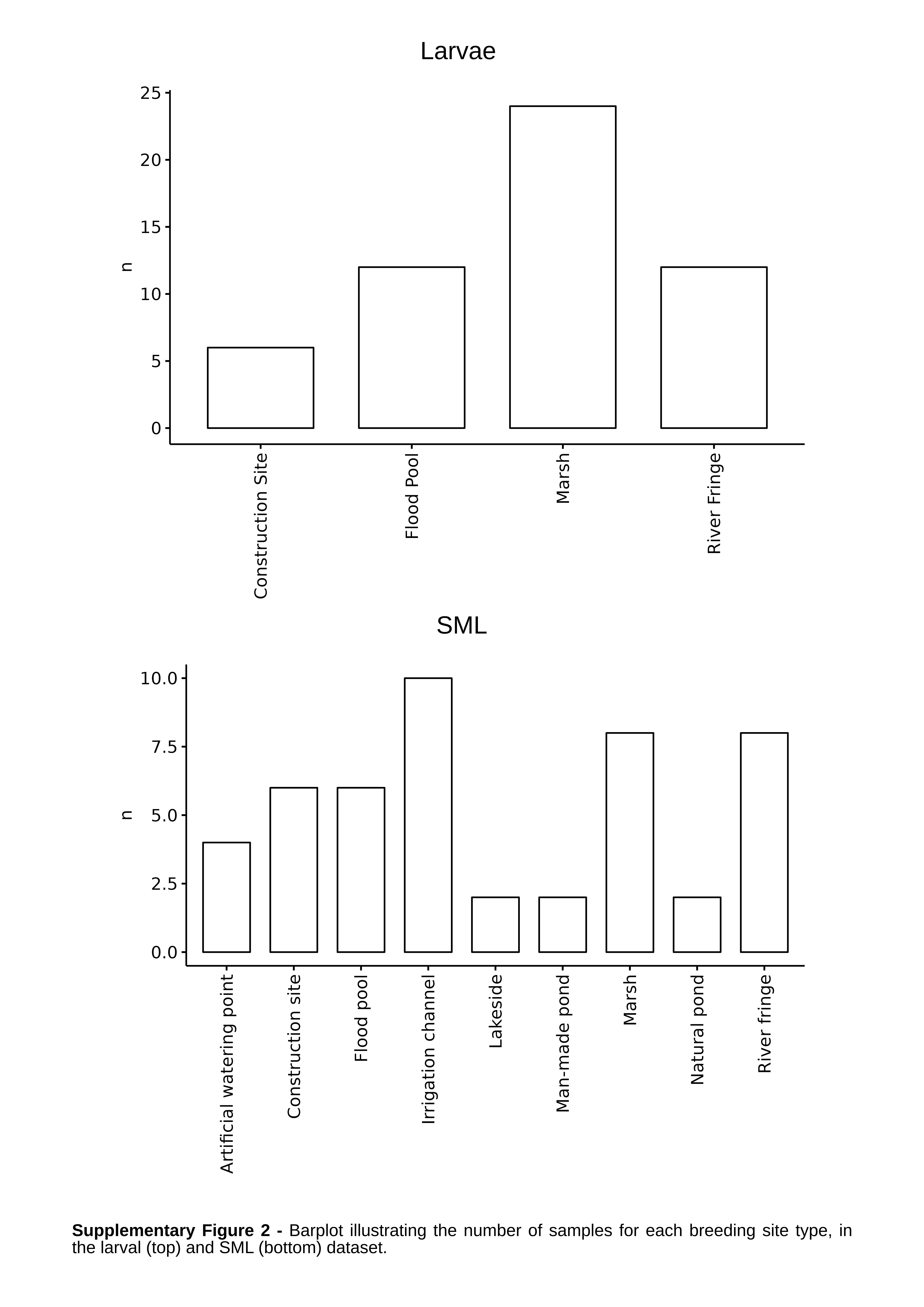

Supplement: fiae161_Supplemental_Files [file fiae161_supplemental_files.zip › supplementary_data-figure_2.jpg]

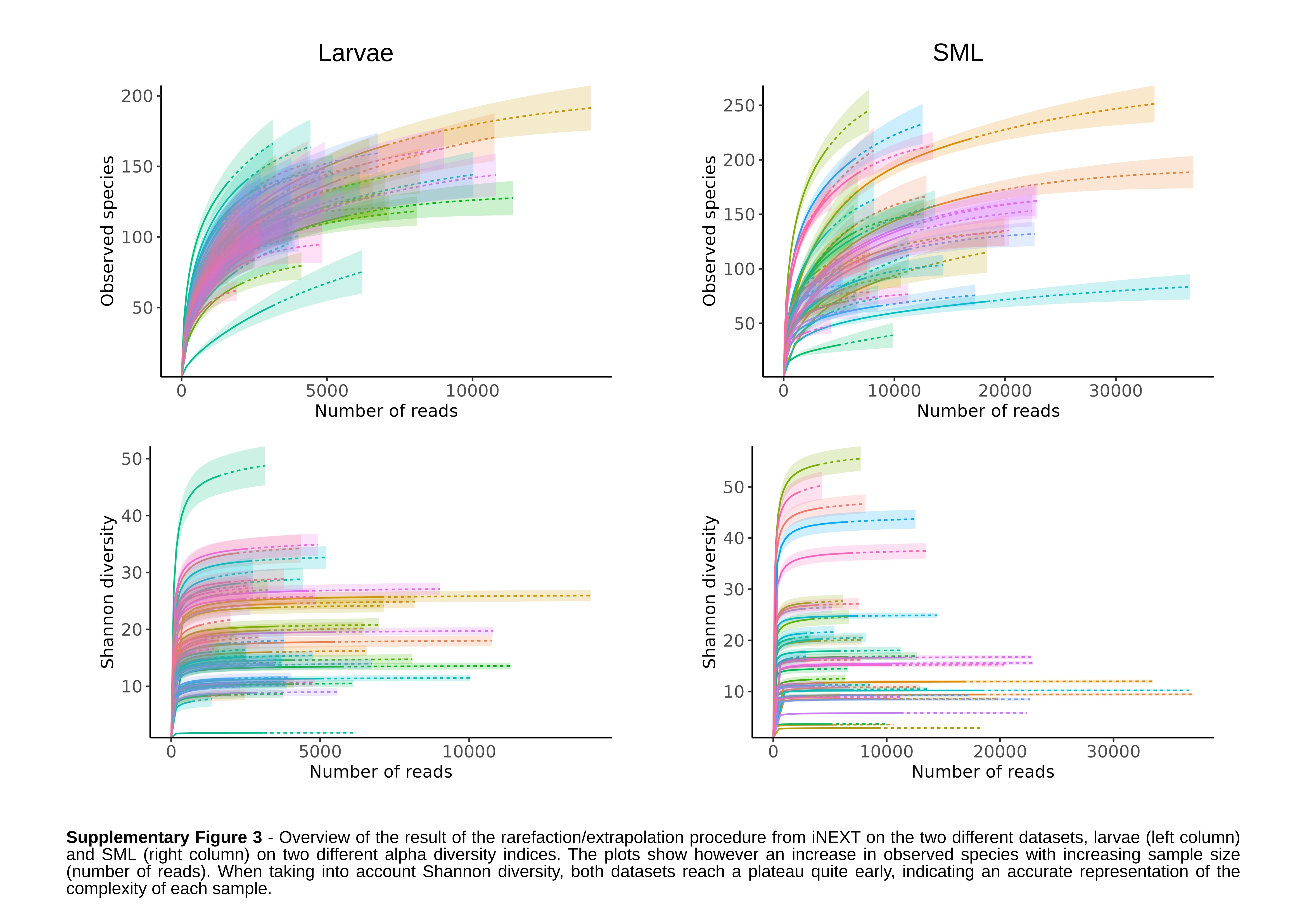

Supplement: fiae161_Supplemental_Files [file fiae161_supplemental_files.zip › supplementary_data-figure_3.jpg]

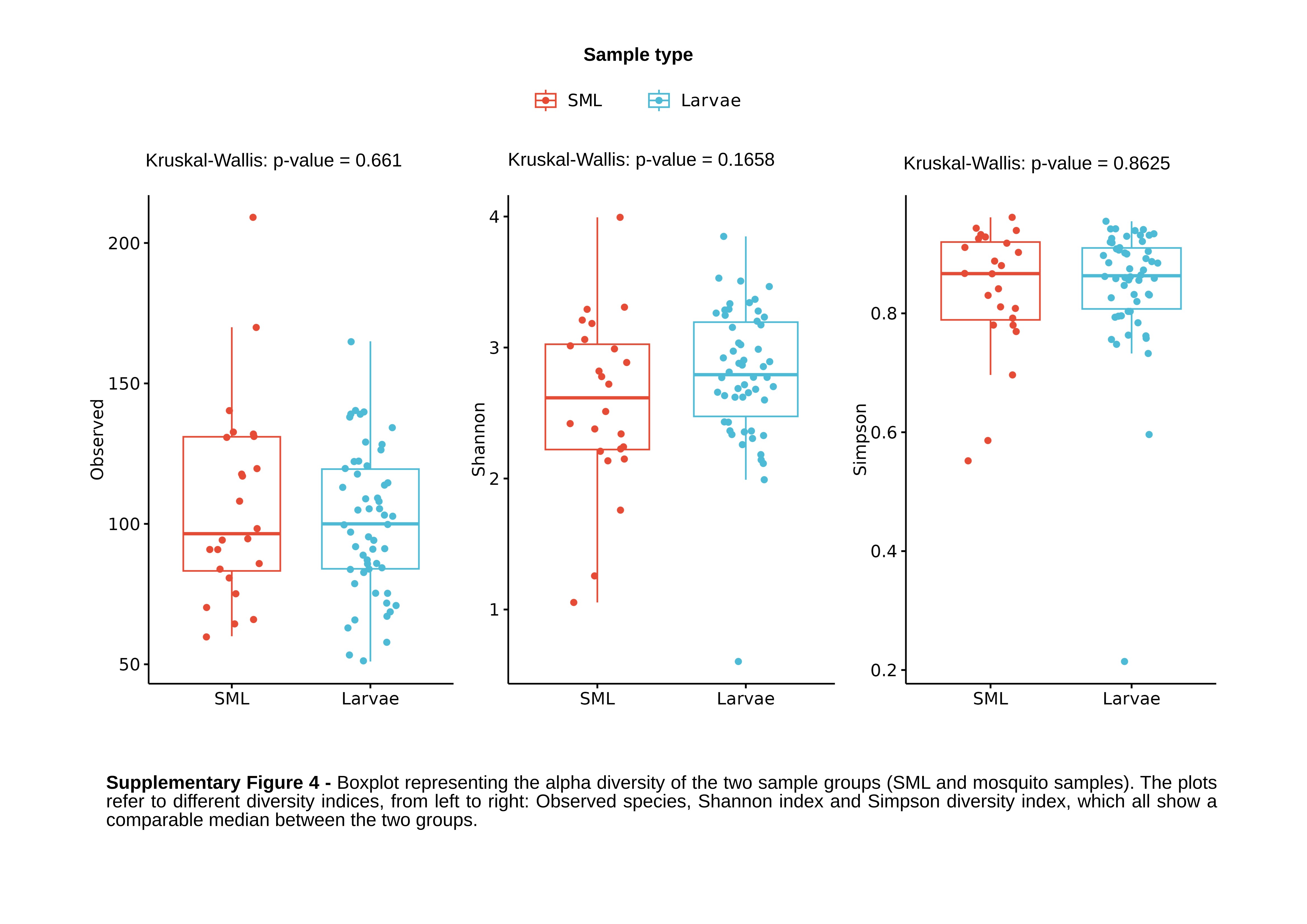

Supplement: fiae161_Supplemental_Files [file fiae161_supplemental_files.zip › supplementary_data-figure_4.jpg]

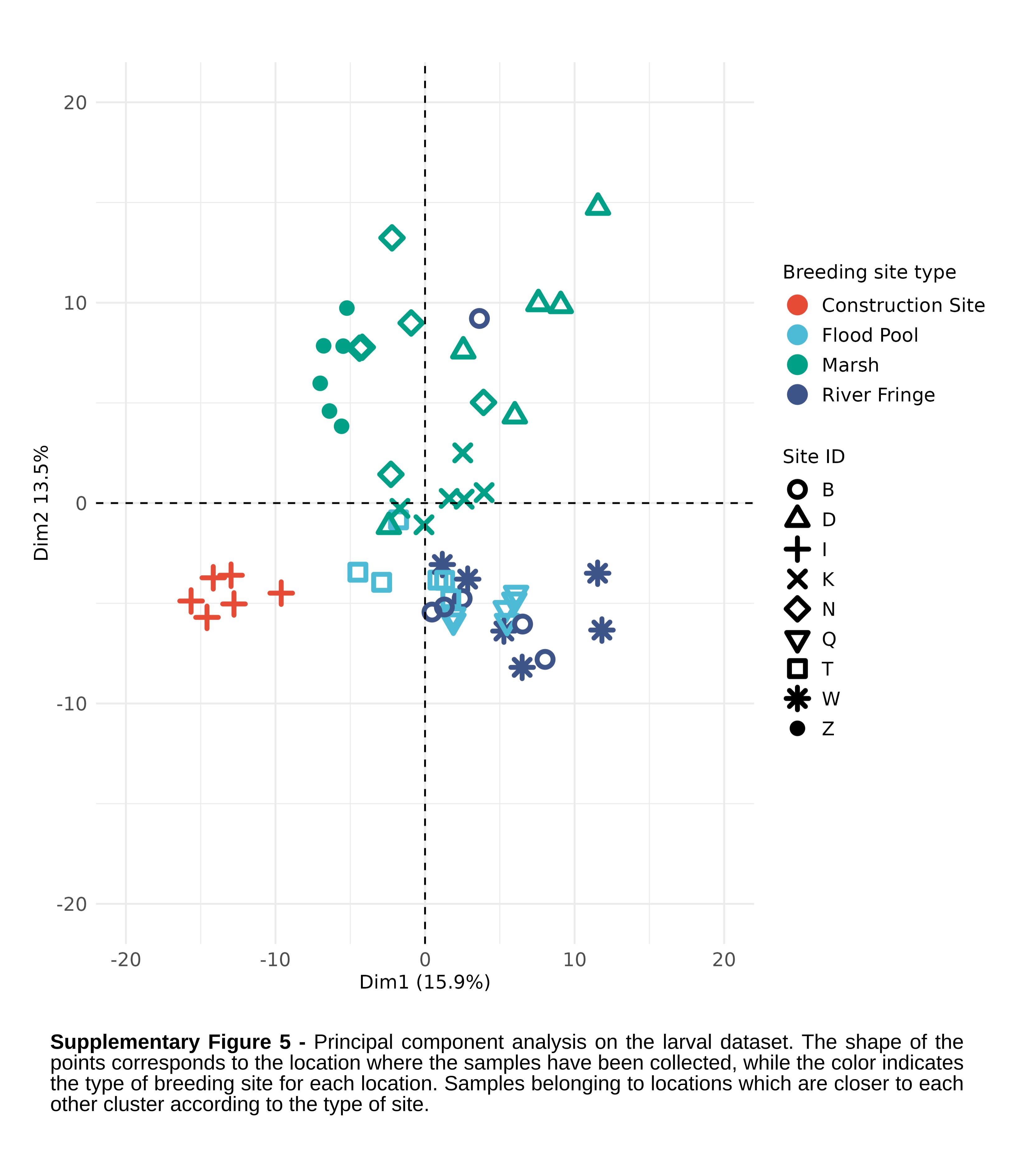

Supplement: fiae161_Supplemental_Files [file fiae161_supplemental_files.zip › supplementary_data-figure_5.jpg]

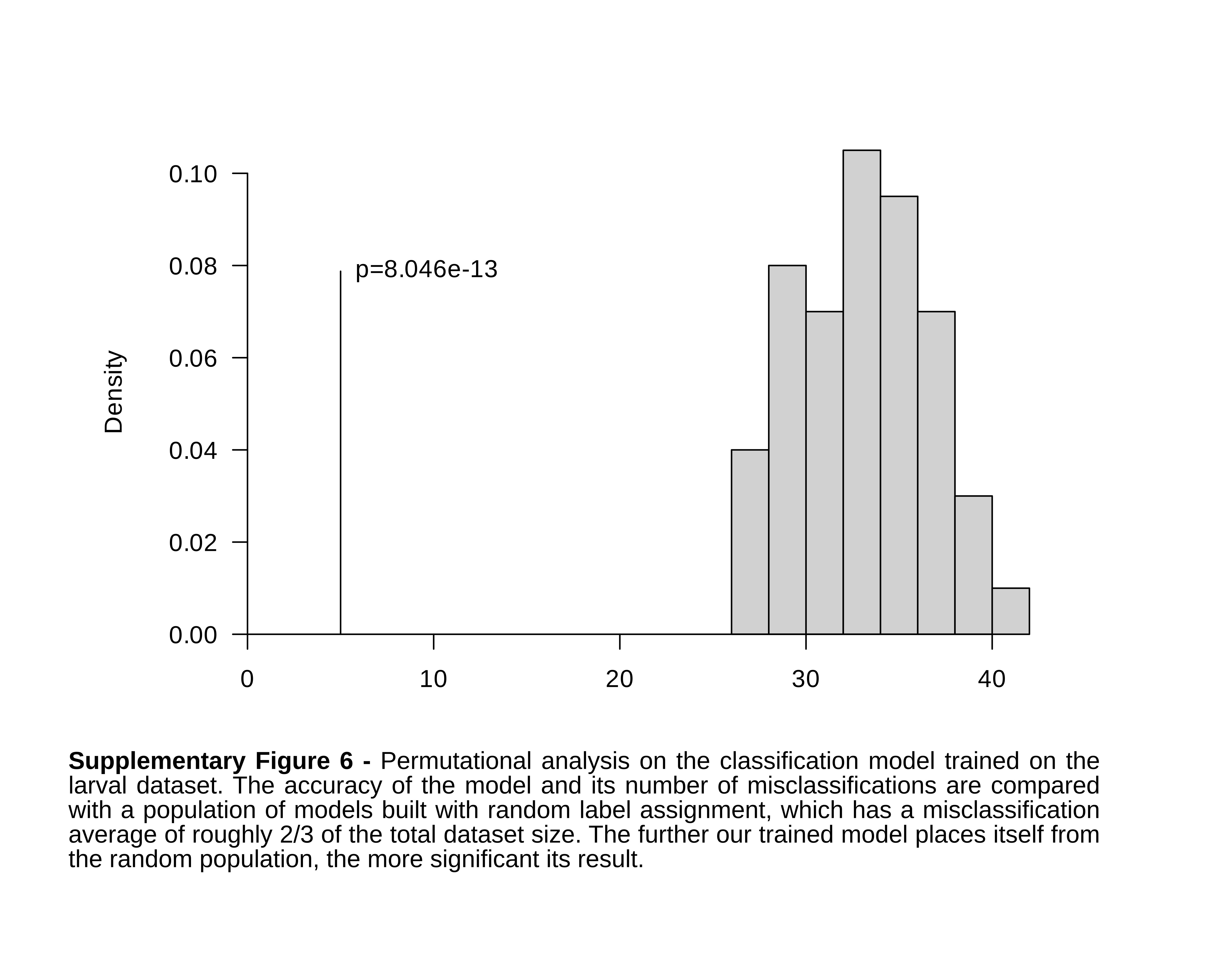

Supplement: fiae161_Supplemental_Files [file fiae161_supplemental_files.zip › supplementary_data-figure_6.jpg]

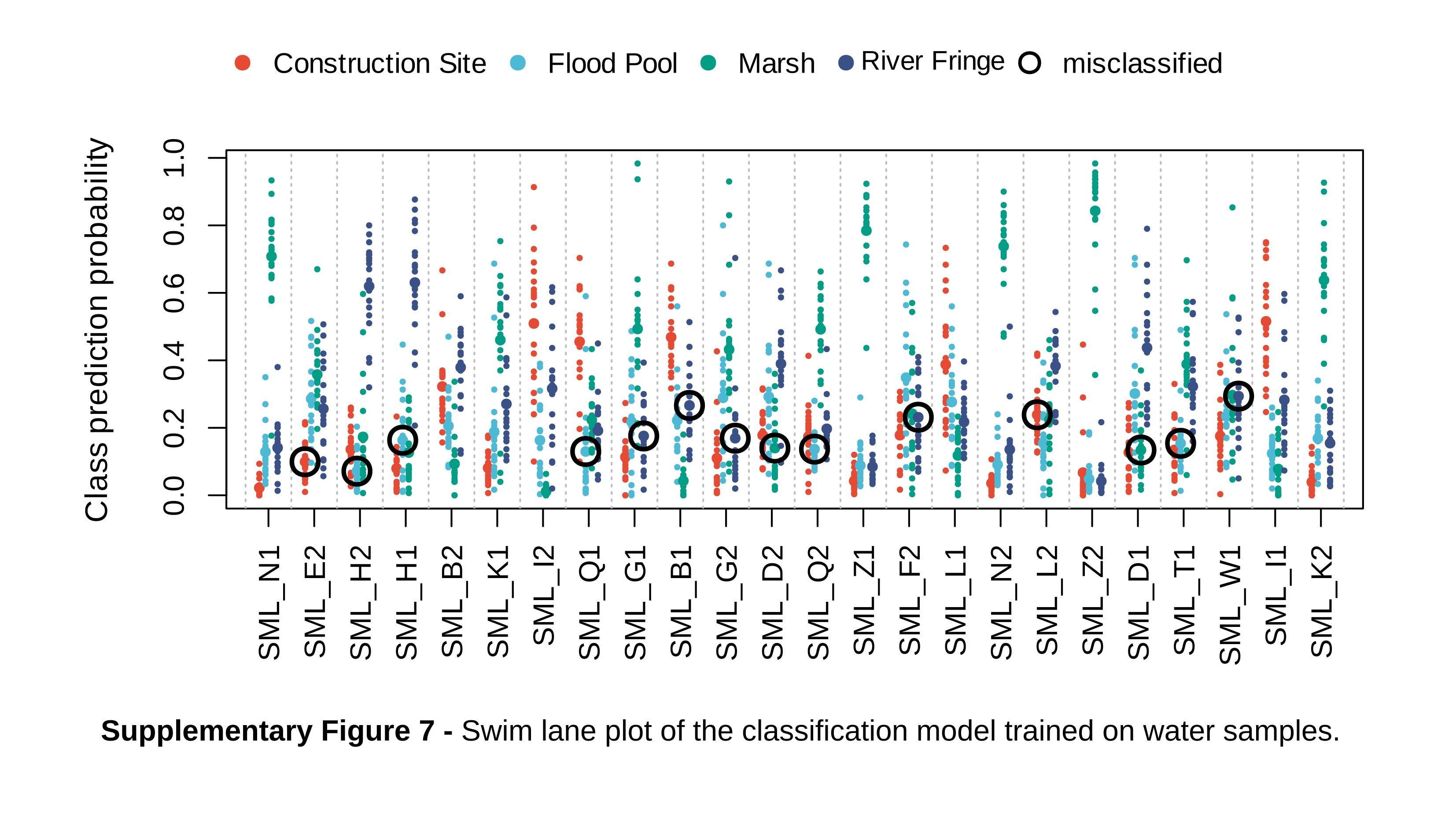

Supplement: fiae161_Supplemental_Files [file fiae161_supplemental_files.zip › supplementary_data-figure_7.jpg]

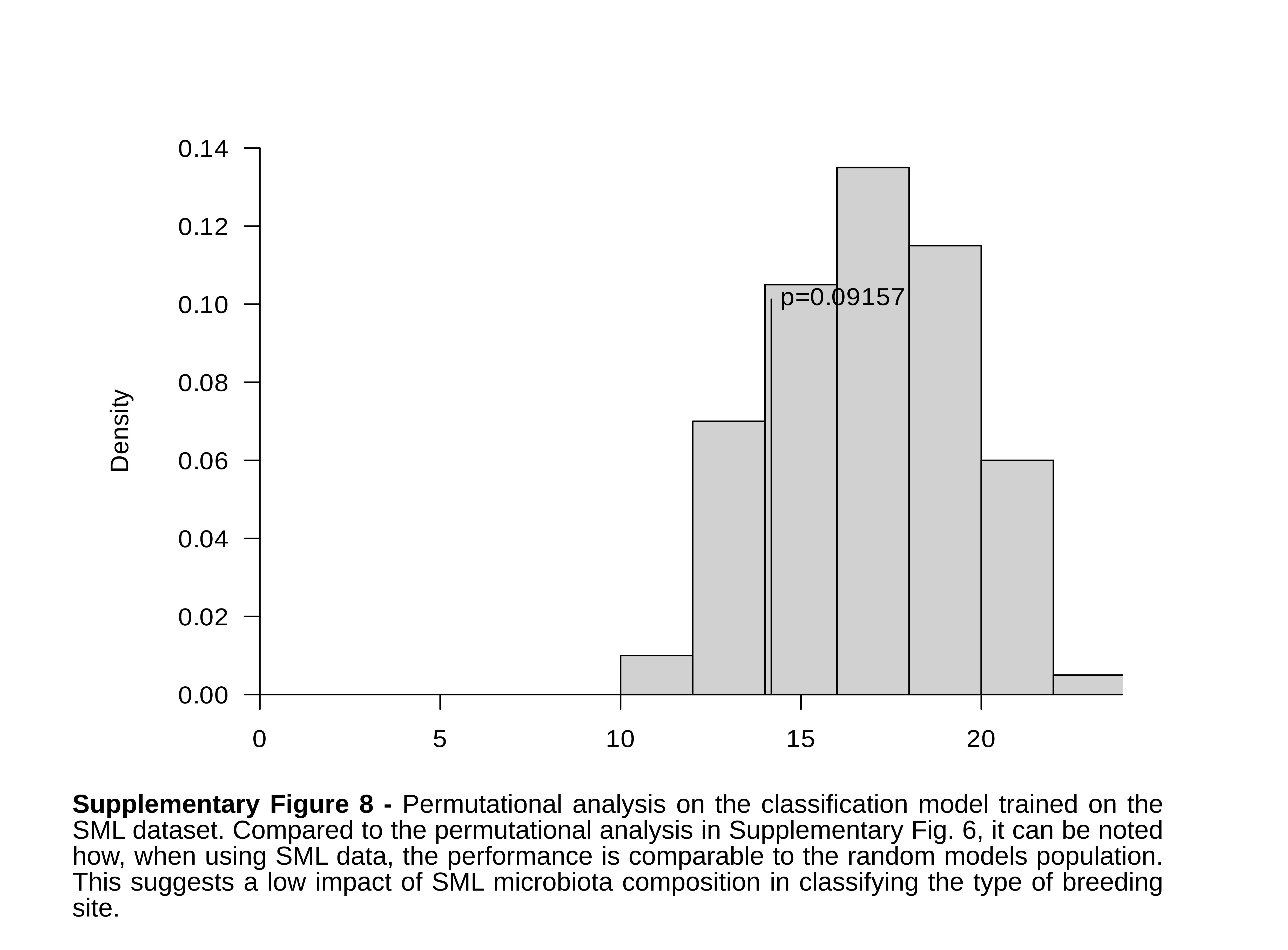

Supplement: fiae161_Supplemental_Files [file fiae161_supplemental_files.zip › supplementary_data-figure_8.jpg]

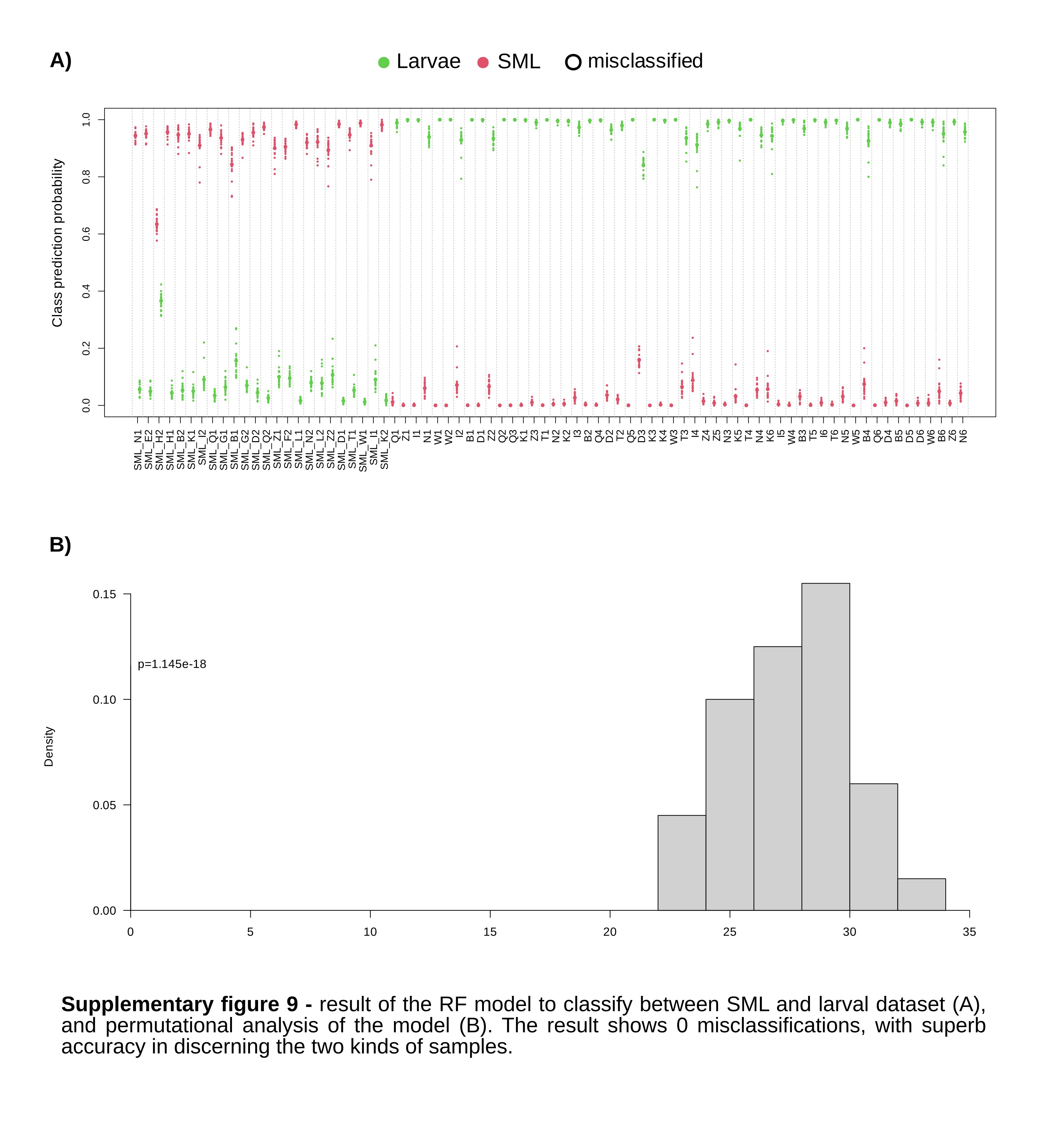

Supplement: fiae161_Supplemental_Files [file fiae161_supplemental_files.zip › supplementary_data-figure_9.jpg]
